# Identification of Genes Underlying Hypoxia Tolerance in *Drosophila* by a P-element Screen

**DOI:** 10.1534/g3.112.003681

**Published:** 2012-10-01

**Authors:** Priti Azad, Dan Zhou, Rachel Zarndt, Gabriel G. Haddad

**Affiliations:** * Department of Pediatrics, University of California-San Diego, La Jolla, California 92093; † Sanford-Burnham Medical Research Institute, La Jolla, California 92037; ‡ Department of Neurosciences, University of California-San Diego, La Jolla, California 92093; § The Rady Children’s Hospital, San Diego, California 92123

**Keywords:** hypoxia, development and survival, Notch pathway, *osa*, *lqf*

## Abstract

Hypoxia occurs in physiologic conditions (*e.g*. high altitude) or during pathologic states (*e.g.* ischemia). Our research is focused on understanding the molecular mechanisms that lead to adaptation and survival or injury to hypoxic stress using *Drosophila* as a model system. To identify genes involved in hypoxia tolerance, we screened the P-SUP P-element insertion lines available for all the chromosomes of *Drosophila*. We screened for the eclosion rates of embryos developing under 5% O_2_ condition and the number of adult flies surviving one week after eclosion in the same hypoxic environment. Out of 2187 lines (covering ∼1870 genes) screened, 44 P-element lines representing 44 individual genes had significantly higher eclosion rates (*i.e.* >70%) than those of the controls (*i.e.* ∼7–8%) under hypoxia. The molecular function of these candidate genes ranged from cell cycle regulation, DNA or protein binding, GTP binding activity, and transcriptional regulators. In addition, based on pathway analysis, we found these genes are involved in multiple pathways, such as Notch, Wnt, Jnk, and Hedgehog. Particularly, we found that 20 out of the 44 candidate genes are linked to Notch signaling pathway, strongly suggesting that this pathway is essential for hypoxia tolerance in flies. By employing the UAS/RNAi-Gal4 system, we discovered that genes such as *osa* (linked to Wnt and Notch pathways) and *lqf* (Notch regulator) play an important role in survival and development under hypoxia in *Drosophila*. Based on these results and our previous studies, we conclude that hypoxia tolerance is a polygenic trait including the Notch pathway.

Whether in pathological conditions or at high altitude, hypoxia can severely affect survival, early development, and fitness of an organism (Mishra and Delivoria-Papadopoulos 1999; Shimoda and Semenza 2011; Webster and Abela 2007). Depending on the duration and severity of hypoxia, cell type, tissue, or organism, the injury caused by hypoxia could be significant and irreversible. Hence, it can result in long-term morbidity and mortality in humans, especially in infants (Ramachandrappa *et al.* 2011). To maintain function and homeostasis, cells sense and respond to inadequate oxygen levels (De Bels *et al.* 2011; Kappler *et al.* 2011; Semenza 2011). Some aspects of the response involve changes in gene expression, and a number of studies have identified various sensitivities of cells and organisms to hypoxic stress (Anderson *et al.* 2009; Clerici and Planes 2009; De Bels *et al.* 2011; Koyama *et al.* 2011; Larson and Park 2009), including a variety of genetic pathways and mechanisms that can potentially affect the response to hypoxia.

Hypoxia-tolerant organisms, such as the African naked mole-rats, Crucian carp, aquatic turtles, and fruit flies, provide a unique opportunity to study the effect of genes influencing hypoxia tolerance or injury *in vivo* (Hochachka *et al.* 1997; Larson and Park 2009; Nilsson and Renshaw 2004). The added advantages of using *Drosophila* as a model system is that their genome has been sequenced, many human disease genes are conserved in *Drosophila*, and a number of genetic tools and stocks are available for manipulation of genes *in vivo*. In particular, there is a vast array of single transposon insertions covering almost the entire *Drosophila* genome (Bellen *et al.* 2004; Spradling *et al.* 1999). We have chosen to perform an unbiased screen of P-Sup P-element lines covering a large portion of the *Drosophila* genome to determine the potentially interesting genes in hypoxia tolerance.

## Materials and Methods

### Fly stocks

P{SUPor-P} (Roseman *et al.* 1995) P-element set for chromosomes X, 2, 3, and Y were obtained from the Bloomington Drosophila Stock Center (Bloomington, Indiana, USA). A list of all the genes included in our P-element screen is attached as supporting information, Table S2. The UAS, TRIP, and RNAi lines were obtained from the Bloomington Drosophila Stock Center and Vienna Drosophila RNAi Center (Vienna, Austria), respectively. *Osa* gene stocks were kindly provided by Dr. Jessica Treisman (NYU School of Medicine). The Gal4 drivers da, Eaat1, Elav, P{GawB}c739, P{GawB}DJ667, He, and Hml were obtained from the Bloomington Drosophila Stock Center.

### P-element screening for hypoxia tolerance

The P-element lines were tested for hypoxia tolerance based on two phenotypes: (1) eclosion rates at 5% O_2_ and (2) adult flies that survived post eclosion at 5% O_2_.

#### Eclosion rates at 5% O_2_:

For each P-element line, 50 females and males were put in a vial with standard corn-meal food. After allowing females to lay eggs for about 6 hr (to obtain about ≥100 eggs), the vials were cleared and the eggs were put under 5% O_2_ for 4 weeks in specially designed computerized chambers (Model A44x0, BioSpherix, Redfield, NY) and ANA-Win2 Software (Version 2.4.17, Watlow Anafaze, CA). After 4 weeks, the number of eclosed and un-eclosed pupae was counted, and the percentage eclosion was calculated for each P-element line tested. Percentage eclosion was determined by calculating the ratio of the number of empty pupae to the total number of pupae in each culture vial. In our screen, we maintained a minimum pupariation of 50% to ensure that the percentage eclosion rate was not biased based on pupae number. We and others have shown that in the *Drosophila* life cycle, the pupal stage is a critical oxygen-sensitive stage, and hence, we chose this phenotype for our screen (Heinrich *et al.* 2011; Peck and Maddrell 2005; Zhou *et al.* 2007). Particularly, we have observed that eclosion under hypoxia for controls is severely affected by hypoxia (eclosion rate less than 10%). The lines that showed percentage eclosion >70% were re-tested at least three times, starting with 100–150 eggs at 5% O_2_, to confirm the results. We chose a 70% cut off since it was significantly higher than all the control fly types (7–8%) and driver fly stocks (45–50%).

#### Adult flies that survived post eclosion at 5% O_2_:

For each line (each P-element line retested as well as controls), we started with 100–150 eggs in the vial and kept them at 5% O_2_ for 4 weeks, and then counted the average number of adults that survived one week after eclosion.

### Real-time PCR analysis of P-element lines

Total RNA was extracted from flies (yw-control and P-elements) under normoxia, using Trizol (Invitrogen, Carlsbad, CA). cDNA was produced from total RNA through RT-PCR using Superscript III First-Strand Synthesis system (Invitrogen).

Real-time PCR was performed using a GeneAmp 7500 sequence detection system using POWER SYBR Green chemistry (Applied Biosystems, Foster City, CA). The expression level of Actin was used to normalize the results (fwd: CTAACCTCGCCCTCTCCTCT; rev: GCAGCCAAGTGTGAGTGTGT). The fold change was calculated using expression level of yw in normoxia as well as hypoxia, which was used as control for all the P-element lines. P-elements with eclosion rate of greater than 85% were tested with real-time PCR. The primer information for the P-elements genes is provided in Table S1.

### Tissue-specific upregulation or downregulation of genes from P-element screen

Depending on the expression of genes in the P-element lines, UAS or RNAi stock of genes were used to overexpress or knockdown the expression of the genes ubiquitously or in specific tissues in the F1 progeny using various Gal4 drivers. The Gal4 drivers used were da (expresses in all tissues), Eaat1 (glial cells), elav-Gal4 [neurons-nervous system (CNS and PNS)], P{GawB}c739 [strong expression in alpha and beta lobe Kenyon cells (intrinsic neurons) of the Mushroom bodies], P{GawB}DJ667 (adult muscles), He-Gal4 (hemocytes), and Hml-Gal4 (larval hemocytes). In the F1 progeny, eclosion rates were calculated after 4 weeks under 5% O_2_ for one developmental cycle (egg-adult). Unpaired Student *t*-tests were used to calculate significant differences in the percentage eclosion of each P-element line, or F1-progeny of UAS, TRIP, or RNAi lines and Gal4 drivers compared with the controls.

### Data analysis and statistical tests

For selection of strongly hypoxia tolerant line we chose a cutoff of >70% eclosion which was 10-fold higher than CS control eclosion rate. The gene ontology (GO)-based analyses were performed using GenMAPP software (Dahlquist *et al.* 2002). The pathway analysis of the candidate genes was done using DAVID program utilizing KEGG and PANTHER, as well as FLIGHT, databases (Huang da *et al.* 2009; Mourikis *et al.* 2010; Saj *et al.* 2010; Sims *et al.* 2006).

## Results

### Genome-wide P-element screen for hypoxia tolerance genes

**Figure 1:**
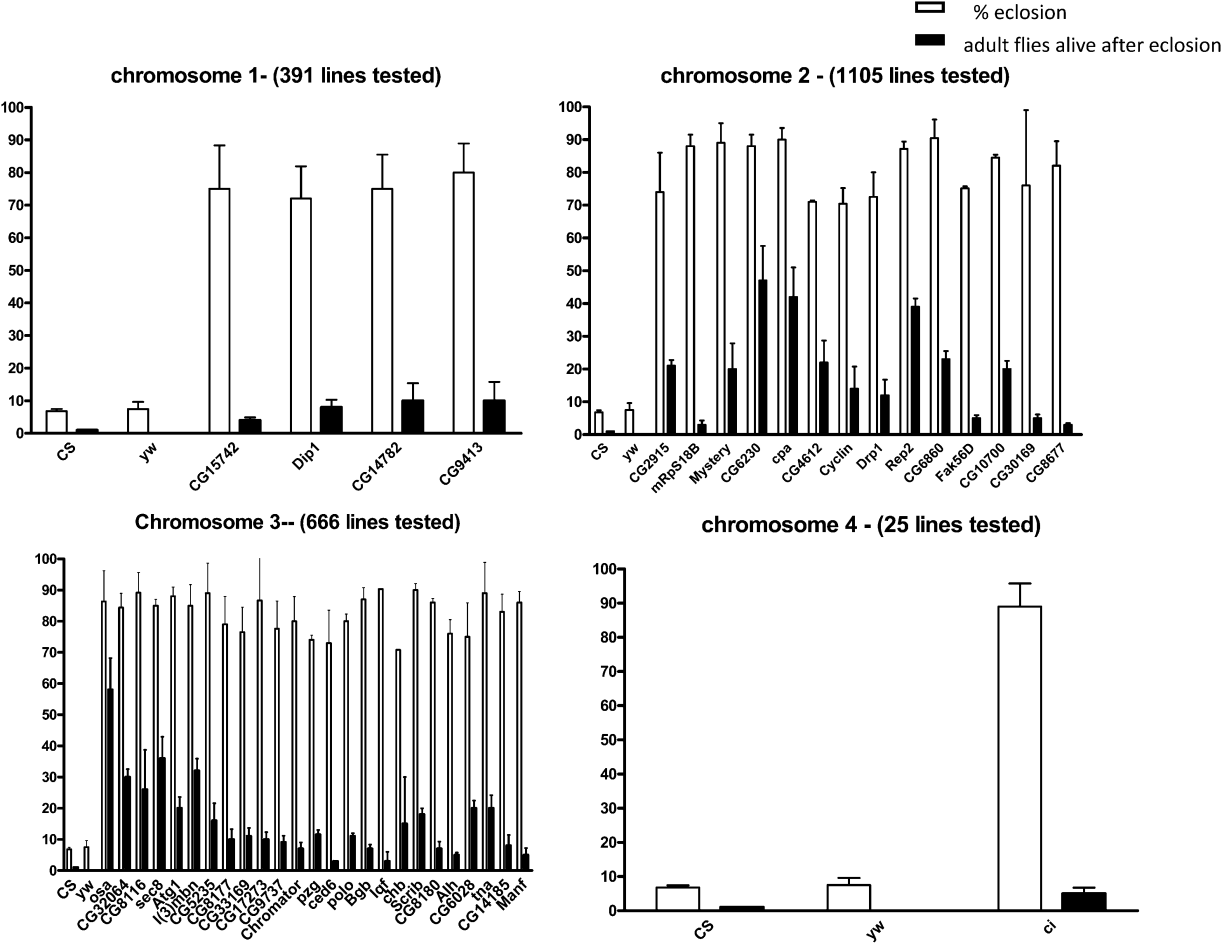
P-element screen for hypoxia tolerance genes. Percentage eclosion and average number of adult flies surviving of P-element lines on chromosomes 1–4 at 5% O_2_. Each bar represents the average of at least three tests for each line (starting with 100–150 eggs); error bars represent the standard error. The number of lines tested for each chromosome is shown in brackets.

To identify genes involved in hypoxia tolerance, we screened P-element insertion lines generated by BDGP Gene Disruption Project (Bellen *et al.* 2004; Roseman *et al.* 1995). We specifically chose SuP or P insertion lines because these P-elements were designed to maximally disrupt genes (Bellen *et al.* 2004; Lukacsovich *et al.* 2001; Roseman *et al.* 1995). Out of 2187 lines (covering ∼1870 genes) screened, 44 P-element lines (44 genes) had rather high eclosion rates (>70% eclosion). Table 1 and Figure 1 show the eclosion rates (each line was retested starting with 100–150 eggs in each vial) and the average number of adult eclosed flies surviving under 5% O_2_ for the P-elements lines that were hypoxia-tolerant. Table 1 also shows the human orthologs of the genes found in our screen. In this screen, we found certain interesting candidate genes, such as *sec8*, *cpa*, *cyclin E*,*osa*, *l(3)mbn*, *Alh*, and *tna*, which show remarkable (70–80%) eclosion rates and hypoxia tolerance during all stages of the developmental cycle (egg to adult) (Table 1 and Figure 1). The eclosion rate of the controls and P-element lines was 98–100%, in normoxia.

**Table 1:** Percentage eclosion and number of adult flies surviving in controls (CS, yw) and P-element lines at 5% O_2_

Gene Symbol	Chr	% Eclosion	Adult Flies	% Pupriation	Molecular Function	Human Orthologs
						Gene Name/Symbol
CS(control)		6.8 ± 0.67	1 ± 0.03	85.7 ± 5.68		
yw(control)		7.5 ± 2.15	0	81.5 ± 10.25		
CG14782	X	75 ± 10.5	10 ± 5.4	97 ± 6.7	Guanyl-nucleotide exchange factor activity	Pleckstrin homology domain containing, family F (with FYVE domain) member 2/ PLEKHF2
CG15742	X	75 ± 13.3	4 ± 0.9	89 ± 10.12	Unknown	
CG9413	X	80 ± 8.9	10 ± 5.8	78 ± 5.15	Amino acid trasmembrane transporter activity	Solute carrier family 7 (glycoprotein-associated amino acid transporter light chain, bo,+ system), member 9/ SLC7A9
Dip1	X	72 ± 9.9	8 ± 2.3	75 ± 3.22	Double-stranded RNA binding	
CG10700	2	84.5 ± 0.95	20 ± 2.5	78 ± 10.2	Electron carrier activity; FAD binding	
CG2915	2	74 ± 12	21 ± 1.8	69 ± 5.67	Metallocarboxypeptidase activity; zinc ion binding	
CG30169	2	76 ± 23	5 ± 1.2	72 ± 12.35	Unknown	
CG4612	2	71 ± 0.45	22 ± 6.7	89 ± 10.42	mRNA binding; poly(A) binding; nucleotide binding	
CG6230	2	88 ± 3.5	47 ± 10.6	82 ± 12.5	ATPase activity, coupled to transmembrane movement of ions, phosphorylative mechanism; ATP binding	ATPase type 13A1/ ATP13A1
CG6860	2	90.47 ± 5.7	23 ± 2.5	80 ± 13.45	Protein binding	Leucine-rich repeats and calponin homology (CH) domain containing 1/ LRCH1
CG8677	2	82.1 ± 7.4	3 ± 0.5	73.1 ± 9.4	Transcription repressor activity; protein binding; zinc ion binding	Cat eye syndrome chromosome region, candidate 2/ CECR2
cpa	2	90 ± 3.6	42 ± 9	85 ± 11.5	Actin binding	Capping protein (actin filament) muscle Z-line, alpha 1/ CAPZA1
CycE	2	70.4 ± 4.8	14 ± 6.8	72 ± 4.2	Cyclin-dependent protein kinase regulator activity	
Drp1	2	72.5 ± 7.5	12 ± 4.8	73 ± 6.77	GTP binding; GTPase activity	Dynamin 1-like/ DNM1L
Fak56D	2	75.19 ± 0.57	5 ± 0.99	72.0 ± 10.22	Protein tyrosine kinase activity	PTK2 protein tyrosine kinase 2/ PTK2
mRpS18B	2	88 ± 3.5	3 ± 1.3	76 ± 11.34	Mitochondrial ribosomal protein, structural constituent of ribosome	Mitochondrial ribosomal protein S18B/MRPS18B
Mys45A	2	89 ± 6	20 ± 7.9	81 ± 12.6	Binding	SDA1 domain containing 1/ SDAD1
Rep2	2	87.2 ± 2.25	39 ± 2.6	75.3 ± 10.27	Protein binding	
Alh	3	76 ± 4.5	5 ± 0.77	75 ± 8.77	Transcription factor activity	Myeloid/lymphoid or mixed-lineage leukemia (trithorax homolog, Drosophila)/ MLLT10
Atg1	3	88 ± 2.99	20 ± 3.6	93 ± 6.90	Protein kinase activity; protein serine/threonine kinase activity; kinesin binding; kinase activity; ATP binding	Unc-51-like kinase 2 (C. elegans)/ ULK2
Bgb	3	87 ± 3.78	7 ± 1.3	77 ± 5.12	Positive regulation of transcription from RNA polymerase II promoter	Core-binding factor, beta subunit/ CBFB
ced-6	3	73 ± 10.6	3 ± 0	83 ± 9.9	Protein binding	GULP, engulfment adaptor PTB domain containing 1/ GULP1
CG14185	3	83 ± 5.66	8 ± 3.44	69 ± 14.65	Protein binding	
CG17273	3	86.7 ± 20.1	10 ± 2.3	82.7 ± 6.8	Adenylosuccinate synthase activity; GTP binding	Adenylosuccinate synthase/ ADSS
CG32064	3	84.4 ± 4.5	30 ± 2.6	80 ± 9.23	Proteolysis	
CG33169	3	76.5 ± 7.99	11 ± 2.7	96.5 ± 10.55	Unknown	
CG5235	3	89 ± 9.7	16 ± 5.6	77 ± 12.6	Dopamine beta-monooxygenase activity	Monooxygenase, DBH-like 1/ MOXD1
CG6028	3	75 ± 10.89	20 ± 2.45	72 ± 9.8	GTP binding	Fumarylacetoacetate hydrolase domain containing 2A/ FAHD2A
CG8116	3	89.2 ± 6.5	26 ± 12.7	92.2 ± 17.5	Unknown	Transmembrane protein 216/ TMEM216
CG8177	3	79 ± 8.97	10 ± 3.33	73 ± 3.2	Anion exchanger activity; inorganic anion exchanger activity	Solute carrier family 4, anion exchanger, member 3/ SLC4A3
CG8180	3	86 ± 1.33	7 ± 2.3	78 ± 7.8	Unknown	
CG9737	3	77.6 ± 8.9	9 ± 2.2	80.6 ± 4.5	Proteolysis; phagocytosis, engulfment	
chb	3	70.8 ± 1.22	15 ± 1.2	90.2 ± 13.75	GTP binding; microtubule binding	Cytoplasmic linker associated protein 1/ CLASP1
Chro	3	80 ± 7.9	7 ± 2	93 ± 6.49	Chromatin binding	
l(3)mbn	3	85 ± 6.79	32 ± 3.9	79 ± 8.5	Plasmatocyte differentiation	
lqf	3	90.3 ± 3.5	3 ± 0.22	93 ± 15.2	Regulation of Notch signaling pathway	Epsin 3/ EPN3
Manf	3	86 ± 3.57	5 ± 2.22	92 ± 9.2	Neuron maintenance; neuron projection development	Mesencephalic astrocyte-derived neurotrophic factor/ MANF
osa	3	86.3 ± 9.9	58 ± 10.2	98.5 ± 10.3	DNA binding; transcription coactivator activity	SWI/SNF
polo	3	80 ± 2.35	11 ± 1	99 ± 10.34	Cell cycle; protein kinase activity	Polo-like kinase 1/ PLK1
pzg	3	74 ± 1.5	11.5 ± 1.5	70 ± 3.67	Cell cycle; establishment or maintenance of chromatin architecture; chromosome organization	
Scrib	3	90 ± 2.1	18 ± 2	86 ± 10.7	Protein binding	
sec8	3	85 ± 2	36 ± 6.9	77 ± 7.89	Neurotransmitter secretion	
tna	3	89 ± 9.86	20 ± 4.22	85 ± 12.5	Chromatin-mediated maintenance of transcription	Zinc finger, MIZ-type containing 2/ ZMIZ2
ci	4	89 ± 6.77	5 ± 1.77	95 ± 8.95	Protein binding, cell cycle regulation	GLI family zinc finger 3/ GLI3

Also shown are human orthologs of the candidate genes.

### Functional categorization of candidate genes

**Figure 2:**
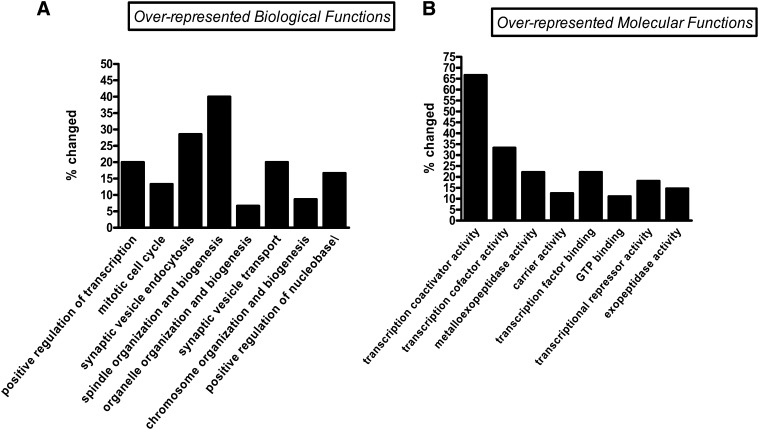
Overrepresented functions in the hypoxia tolerant P-element lines as computed by GO. (A) Biological processes predominant for hypoxia tolerance (egg-adult) under 5% O_2_. (B) Molecular processes predominant for hypoxia tolerance (egg-adult) under 5% O_2_.

GO and pathway analyses were performed to determine the predominant biological processes and pathways that are potentially regulated by the candidate genes and play a role in hypoxia tolerance. The biological process categories in which these candidate genes were overrepresented include spindle organization, synaptic vesicle endocytosis and transport, regulation of transcription, and cell cycle (Figure 2A). The molecular function of the mutated genes in the hypoxia-resistant P-element insertion lines ranged from transcriptional co-regulators, to DNA or protein binding, to ATP and GTP binding, to carrier activity, to metalloexopeptidase and exopeptidase activity (Figure 2B and Table 1). For example, we found that P-element lines of a number of transcriptional regulators, such as *osa*, *Alh*, and *tna*, had a strong hypoxia resistance phenotype. Starting with 100–150 eggs, we observed that the downregulated osa line had a high eclosion rate (86%) and that the average number of flies that survived after eclosion are ∼58 flies (>50%), which is significantly higher compared with controls (eclosion rate 7%, and average number of adults surviving after eclosion <2). Table 2 shows the pathways related to the 44 candidate genes found in our screen. It is intriguing that we find a strong link to Notch pathway (20 genes/44 genes), but at the same time, we also discovered other pathways, such Wnt, Erk, Hedgehog or JAK/ STAT, and VEGF pathways, that seem to play important roles in hypoxia tolerance.

**Table 2:** Signaling pathways linked to the candidate genes

Symbol	Gene	Signaling Pathway*^a^*
CG15742	CG15742	JNK modifier
Dip1	CG15367	1) Innate immunity 2) Notch signaling
CG14782	CG14782	1) JNK modifier 2) Notch signaling
CG9413	CG9413	Not detected
CG2915	CG2915	Not detected
mRpS18B	CG10757	Notch signaling
Mys45A	CG8070	1) Lipid storage 2) Notch signaling 3) Cardiogenic genes
CG6230	CG6230	Notch signaling
cpa	CG10540	1) M. fortuitum infection 2) Morphogenesis 3) Phagocytosis
CG4612	CG4612	1) JNK modifier 2) Mito Ca^2+^ and H+ regulation
CycE	CG3938	1) M. fortuitum infection 2) Morphogenesis 3) Lipid storage 4) miRNA pathway 5) cell cycle 6) p53 pathway 7) Ubiquitination pathway
Drp1	CG3210	1) Mito morphology 2) Notch signaling 3) Ca^2+^ signaling (Ca^2+^ entry) 4) Endocytosis
Rep2	CG1975	Notch signaling
CG6860	CG6860	Not detected
Fak56D	CG10023	1) Angiogenesis 2) Integrin signaling pathway 3) VEGF signaling pathway
CG10700	CG10700	Not detected
CG30169	CG30169	Not detected
CG8677	CG8677	Not detected
osa	CG7467	1) Wnt signaling 2) Mito Ca^2+^ and H+ regulation 3) Notch signaling
CG32064	CG32064	1) Glutathione metabolism 2) Sesquiterpenoid and triterpenoid biosynthesis in Urea cycle metabolism
CG8116	CG8116	Notch signaling
sec8	CG2095	1) *E. coli*/*S. aureus* infection 2) Phagocytosis
Atg1	CG10967	1) Cell cycle kinase 2) Notch pathway 3) Regulation of authophagy 4) mTOR signaling pathway
l(3)mbn	CG12755	ERK signaling
CG5235	CG5235	Not detected
CG8177	CG8177	1) Multipolar division 2) Ca^2+^ signaling (Ca^2+^ entry inhibition)
CG33169	CG33169	Notch signaling
CG17273	CG17273	1) Innate immunity 2) Purine metabolism 3) Alanine-aspartate and glutamate metabolism 4) Wnt signaling pathway 5) De novo purine biosynthesis 6) Metabolic pathways
CG9737	CG9737	Phagocytosis
Chro	CG10712	1) M. fortuitum infection 2) Hedgehog signaling 3) Notch signaling
pzg	CG7752	1) JAK/STAT signaling 2) ERK signaling 3) E2F signaling 4) Notch signaling 5) Hedgehog signaling 6) M. fortuitum infection 7) Ca^2+^ signaling
ced6	CG11804	1) *C. trachomatis* infection 2) Ca^2+^ signaling
polo	CG12306	1) Cell cycle kinase 2) Kinase cell progression 3) Centrosome number 4) Mitosis 5) Morphogenesis 6) Cytoskeletal morphogenesis 7) DFoxO signaling 8) Notch signaling 9) Phagocytosis 10) Apoptosis pathway 11) Progesterone-mediated oocyte maturation 12) Endocytosis
Bgb	CG7959	Not detected
Iqf	CG8532	1) Insect dengue virus infection 2) Endocytosis 3) Notch signaling
chb	CG32435	1) ERK signaling 2) Tublin flux 3) Mitosis
Scrib	CG42614	1) Innate immunity 2) Cardiogenic genes 3) Notch signaling 4) Ca^2+^ signaling
CG8180	CG8180	1) JAK/STAT signaling 2) ERK signaling
Alh	CG1070	1) Cell growth and viability 2) Mito Ca^2+^ and H+ regulation 3) Notch signaling
CG6028	CG6028	Not detected
tna	CG7958	1) Cell growth and viability 2) Wnt signaling 3) Notch signaling 4) Hedgehog signaling 5) Ca^2+^ signaling 6) Dpp signaling 7) Interferon-gamma signaling pathway 8) JAK/STAT signaling pathway
CG14185	CG14185	Notch signaling
Manf	CG7013	Not detected
ci	CG2125	1) Hedgehog signaling 2) Notch signaling

a Signaling pathways are based on DAVID (KECK and PANTHER database) and FLIGHT database.

### Overexpression or knockdown of single genes and hypoxia tolerance

**Figure 3:**
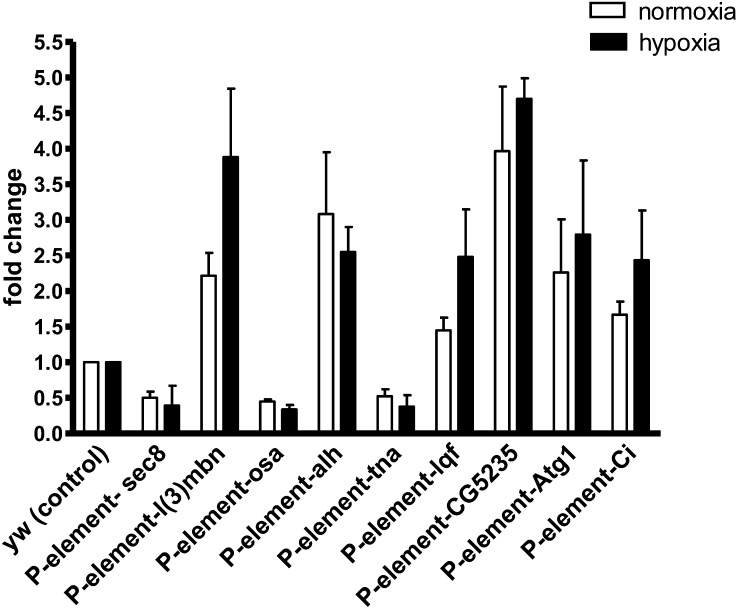
Gene expression in P-element lines. Real-time PCR analysis of P-element lines in normoxia and hypoxia. Means are statistically significant when *P* < 0.05 (unpaired *t*-test comparing P-element lines with yw control).

**Figure 4:**
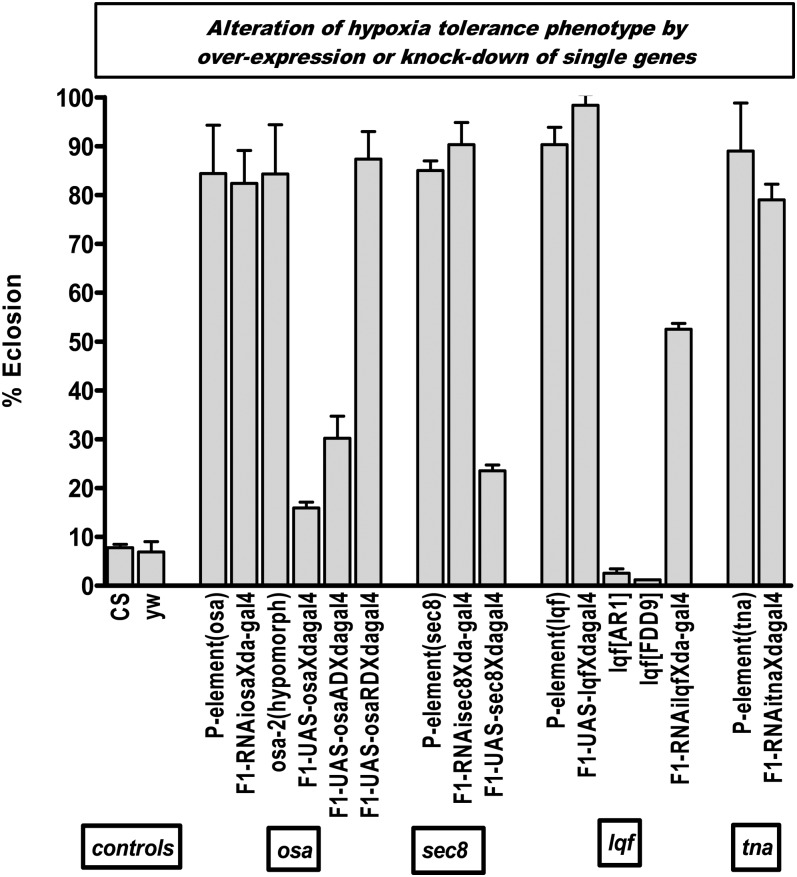
Effect of alterations of single genes on hypoxia tolerance phenotype. Percentage eclosion of flies in which single genes were overexpressed or knocked out based on the real time PCR analysis of P-element lines. Each bar represents the average of at least three tests for each line (starting with 100–150 eggs); error bars represent the standard error.

Before we studied the role of each differentially expressed gene, we performed real-time PCR, as shown in Figure 3. PCR showed that in these P-elements, the expression of some of the genes was indeed significantly altered under normoxia and hypoxia (Figure 3). For example, *sec8*, *osa*, and *tna* were significantly downregulated, and *l(3)mbn*, *Alh*, *lqf*, *CG5235*, *atg1*, and *ci* were more than 1.5-fold upregulated. To understand the mechanisms underlying hypoxia tolerance *in vivo*, we overexpressed (using the UAS-Gal4 system) or knocked down (RNAi) these genes ubiquitously (*e.g*. da-gal4 drivers) or in specific tissues, depending on whether these particular genes were upregulated or downregulated in the P-elements (Figure 4). We chose to study in detail 4 genes (out of the 44 from our initial screen) based on the following criteria: a) they showed a strong hypoxia phenotype [*e.g.* the *osa* gene had a percentage eclosion of 86.3 ± 9.9 and had the highest average number of flies (58 ± 10.2) that survived post eclosion]; b) they showed a clearly significant upregulation or downregulation in the P-element line by real-time PCR; and c) availability of fly lines (either UAS or RNAi) and mutants to further study their effect *in vivo*. Hence, we decided to further study the following genes: *osa*, *lqf*, *tna*, and *sec8* (Figure 4). Indeed we found that the upregulation or downregulation of these genes in these P-element lines had a functional significance under hypoxia. When we upregulated or knocked down the genes using UAS, TRIP, or RNAi lines and ubiquitous da-GAL4 driver, the resulting F1 progeny and mutant stocks matched the phenotype we observed in the P-element lines under hypoxia. For example, we found that knockdown of *osa* (either by a TRIP RNAi or with a hypomorph mutant) leads to a tremendous increase of eclosion of flies in hypoxia (*P* < 0.05; Figure 4). We also tested the artificial constructs of *osa* gene in which the gene was attached to a constitutive activating or repressor domain (Collins *et al.* 1999). Our results showed that constitutive repression of osa (as seen in F1-UAS-osaRDXdagal4) leads to better eclosion under hypoxia, whereas upregulation of osa (F1-UAS-osaXdagal4 or F1-UAS-osaADXdagal4) leads to significantly lower eclosion rate under hypoxia. This is consistent with the hypothesis that knockdown or loss of osa expression leads to significantly better eclosion of flies at 5% O_2_, indicating that osa is a repressor of genes that are important in hypoxia tolerance. Similarly, we found that an *in vivo* loss of *sec8* and *tna* function gives a survival advantage for eclosion in 5% O_2_. In contrast, an upregulation of the *lqf* gene (F1-UASlqfXdagal4) significantly increases (98% eclosion compared with controls with eclosion rate of 7%) the eclosion rate of flies under hypoxia. Knockdown of *lqf* (in mutant stocks lqfAR1, FDDR9, and F1-TRIP RNAiXda-gal4) tremendously reduced eclosion rates (Figure 4). This is very intriguing as *lqf* is a Notch regulator, and we have previously shown the importance of Notch in hypoxia adaption in flies (Zhou *et al.* 2011).

### Tissue-specific overexpression or knockdown of *osa* and *lqf* genes

**Figure 5:**
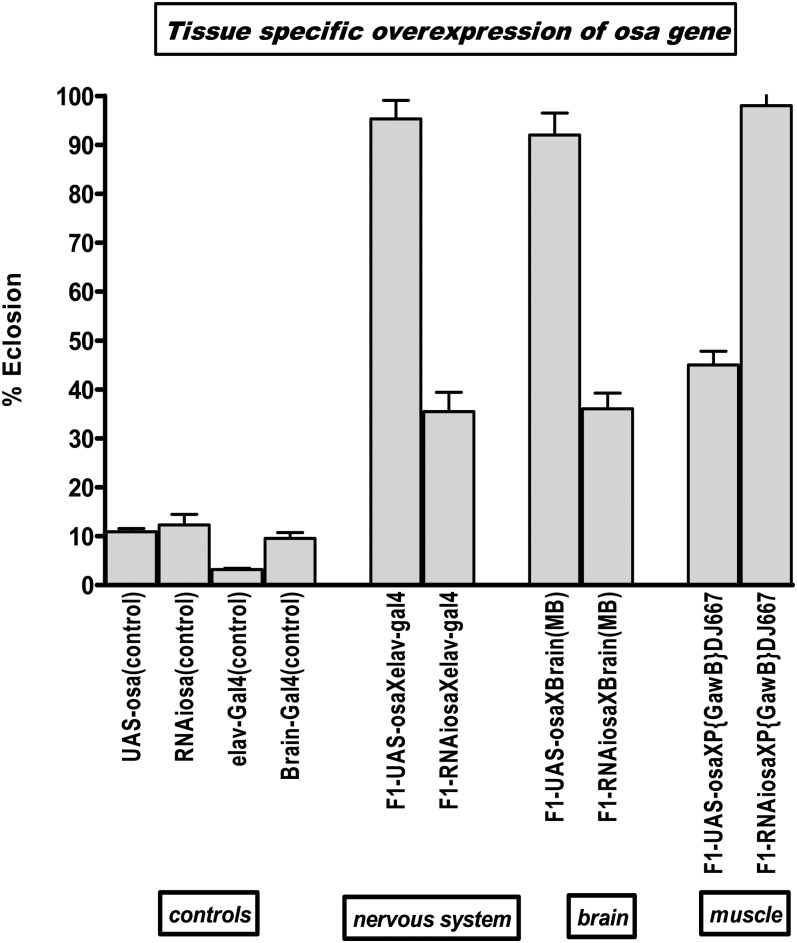
Effect of tissue-specific overexpression of *osa*. *Osa* was upregulated in specific tissues using Gal4 drivers: (elav-gal4) nervous system, (c736) mushroom body of the brain, and (P{GawB}DJ667) muscle driver. The figure shows percentage eclosion of F1 progeny of the crosses. Each bar represents the average of at least three tests for each line (starting with 100–150 eggs); error bars represent the standard error.

To determine whether there is any tissue-specific effect of knockdown or overexpression in various tissues such as the central nervous system, we utilized progenies of crosses made with specific GAL4 drivers. We then subjected the F1 progeny of such crosses to 5% O_2_ and quantified eclosion rates. As depicted in Figure 5, our data show that the specific knockdown of osa in the nervous system (elav-gal4) and mushroom body (MB) of the brain has an opposite effect on eclosion, as compared with increasing its expression ubiquitously (*i.e.* its knockdown in these tissues decreased eclosion rates) (Figures 4 and 5). This suggests that osa has a specific role in the central nervous system and that under hypoxia its loss of function decreases eclosion rates. Knockdown of *osa* using the muscle-specific driver shows a similar phenotype of strong eclosion rate (90%) as ubiquitous expression (Figures 4 and 5)

**Figure 6:**
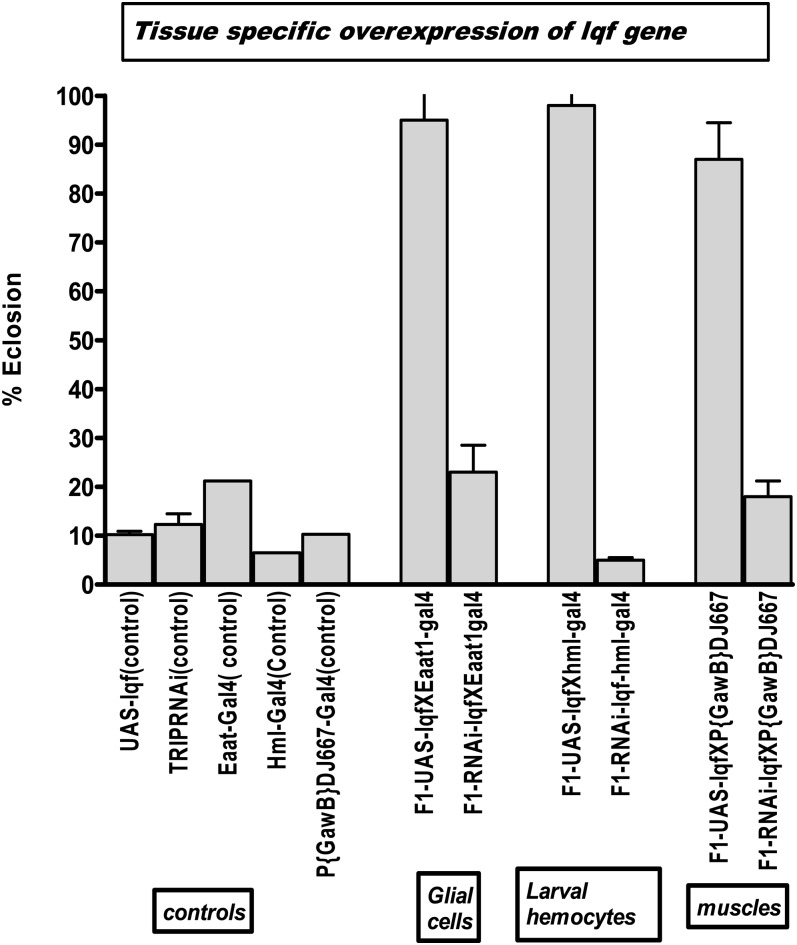
Effect of tissue-specific overexpression of lqf. lqf was upregulated in specific tissues using Gal4 drivers: (Eaat1) glial cells, (Hml-Gal4) larval hemocytes, and (P{GawB}DJ667) muscle driver. The figure shows percentage eclosion of F1 progeny of the crosses. Each bar represents the average of at least three tests for each line (starting with 100–150 eggs); error bars represent the standard error.

Figure 6 shows data related to the *lqf* gene. We have observed that upregulation of *lqf* in glial cells leads to a significantly higher eclosion (93%, *P* < 0.001). Furthermore, specific upregulation of *lqf* in larval hemocytes increased eclosion (99%, *P* < 0.001 *vs.* controls), and its knockdown had an opposite effect. Under 5% O_2_, knockdown of *lqf* specifically in the muscles tremendously reduces eclosion rates. This may be linked to the abnormalities in wings and legs caused by loss of *lqf* expression (Cadavid *et al.* 2000), but we do not observe any significant lowering of eclosion rates under normoxia. This suggests that under hypoxia, the knockdown of *lqf* in muscles has a significant impact on development.

## Discussion

In the present study, we used a genome-wide P-SUP transposable element screen for hypoxia tolerance during all developmental stages in flies, starting from embryos at 5% O_2_. Out of 1870 genes screened, 44 genes showed strong hypoxia tolerance phenotype. This is intriguing because this is a relatively small number of genes that show a relation to hypoxia, indicating that there is some specificity between phenotype and genotype. This phenotype of hypoxia tolerance of these P-element lines was strong as they did not only show increased eclosion rate but also the number of flies that survived after eclosion was impressive compared with the wild-type flies. This result indicates that these candidate genes not only help in hypoxia tolerance across development but also in the adult after eclosion. We have examined the role of *sec8*, *osa*, *tna*, and *lqf* genes in hypoxia tolerance *in vivo*. These genes have varied molecular and biological functions but have not been previously studied in the context of survival in hypoxia. For instance, sec8 is a part of an evolutionarily conserved eight-subunit protein complex that is required for tethering exocytic carriers to target membranes in eukaryotic cells (Oztan *et al.* 2007). The liquid facets locus (*lqf*) encodes epsin, a vertebrate protein associated with the clathrin endocytosis complex (Cadavid *et al.* 2000). Recent studies support the view that many proteins governing membrane sorting during endocytosis participate also in nuclear signaling and transcriptional regulation, mostly by modulating the activity of various nuclear factors (Pyrzynska *et al.* 2009). A number of these proteins are implicated in the regulation of cell proliferation and tumorigenesis (Pyrzynska *et al.* 2009). In addition, besides endocytosis, sec8 is also involved in the regulation of synaptic microtubule formation and glutamate receptor trafficking (Liebl *et al.* 2006). Hence, these genes through their endocytic, neuronal, or transcriptional regulatory function significantly help in hypoxia tolerance.

*Osa* gene may also be acting as a transcriptional regulator. Indeed it is genetically linked to three other genes found in our present screen (*i.e. CycE*, *Alh*, and *tna*) (Baig *et al.* 2010; Gutierrez *et al.* 2003). Recent studies have suggested an intriguing role for osa, which is to establish a chromatin environment in the regulatory regions of EGFR as well as WNT target genes, making them available for both activators and repressors and facilitating transcription in response to signaling (Collins and Treisman 2000; Terriente-Felix and de Celis 2009). Osa-containing Brahma chromatin remodeling complexes are required for the repression of wingless target genes (Collins *et al.* 1999; Collins and Treisman 2000; Treisman *et al.* 1997). This osa-mediated repression acts on Groucho/Pangolin complex and specific downstream genes, such as Dpp, nubbin, and ubx of the Wg pathway (Collins *et al.* 1999; Collins and Treisman 2000; Lopez *et al.* 2001; Vazquez *et al.* 1999). It is also noteworthy that osa showed tissue specificity, as its effect in the nervous system is opposite to that when it is expressed ubiquitously. A previous study has shown that *osa* can negatively regulate proneuronal development through *pannier* and *chip* genes through chromatin remodeling (Heitzler *et al.* 2003). Hence, we can infer that it can act both as a positive and negative regulator of transcription, depending on its location and physiological function.

In previous studies in our laboratory, we have shown an effect of Notch on survival and adaptation of flies selected over generations under hypoxia (Fan *et al.* 2005; Gustafsson *et al.* 2005; Zhou *et al.* 2011). Interestingly, in this study, we also find genes linked to Notch pathway as shown in Table 2. This is remarkable as there is no *a priori* reason for the screen to be baised to one pathway or another. Besides, in our current study no selection or adaptation to long-term hypoxia has been utilized. Nevertheless, a link to the Notch pathway for hypoxia tolerance during one generation is very interesting and would indicate that the Notch pathway is not only important for hypoxia survival in long-term (transgenerational) conditions but also in shorter-term hypoxia, including during development.

It is known that *osa* and *lqf* are strongly linked to the Notch pathway (Armstrong *et al.* 2005; Kankel *et al.* 2007; Vaccari *et al.* 2008; Windler and Bilder 2010). In fact, lqf (ortholog of Mammalian Epsin) is a Notch regulator through Delta (Overstreet *et al.* 2004). Epsin modulates Notch pathway activity in *Drosophila* and *C. elegans* (Tian *et al.* 2004). It interacts with the Notch pathway during multiple Notch-dependant events in *Drosophila* (Tian *et al.* 2004). Ligands of the Delta and Serrate must normally be endocytosed in signal-sending cells to activate Notch (Overstreet *et al.* 2004; Wang and Struhl 2005). It has been shown that only those molecules of Ser and Dl that are targeted by ubiquitination to enter the Epsin (vertebrate lqf)-dependent pathway have the capacity to activate Notch (Todi and Paulson 2011; Wang and Struhl 2005). Genetic studies have shown that the BRM complex (composed of brm, osa, and moira) shows a close functional connection with Notch signaling (Armstrong *et al.* 2005). Hence, these genes could be functioning through the Notch signaling pathway to provide strong hypoxia tolerance. For example, osa is known to affect wing tissue, independent of its effect on the Wnt pathway. This might be related to Notch signaling in these cells as osa is also required to promote Dl (Notch ligand) expression in vein territories (Terriente-Felix and de Celis 2009). In addition, through its chromatin-remodeling activity, osa is known to regulate the cell cycle by coordinating cell-cycle progression through downstream genes, such as CycE interaction or string/cdc25 expression, in normal *vs.* cancer cells (Baig *et al.* 2010; Brumby *et al.* 2002; Moshkin *et al.* 2007). This cell-cycle arrest of cells requires the function of several signaling pathways: Wg, Egfr, and Notch as well as chromatin-remodeling controlling cell proliferation through the Notch pathway. Indeed, in our screen we found that P-element lines affecting CycE as well as Alh (polycomb gene controlling chromatin-structure) also had strong eclosion under hypoxia. This might be linked to Osa-CycE interaction or Osa-Alh chromatin modeling mediated by Notch regulation (Saj *et al.* 2010). To study the effect of CycE overexpression in proliferation of bristle lineage in *Drosophila*, Simon *et al.* (2009) have shown that Notch acts as a repressor, whereas in Scrib mutants, Notch aids cooperatively in cell proliferation and survival with the Scrib gene (Brumby and Richardson 2003). The Notch signaling pathway and its interaction with ATG1 may be related to the function of Notch in macroautophagy during fly metamorphosis (Kiffin *et al.* 2006). In a recent study, it has been shown that in *Drosophila* crystal cells, HIF1α/sima activates Notch receptor signaling, which promotes hemocyte survival during both normal hematopoietic development and hypoxic stress (Mukherjee *et al.* 2011). Hypoxia-inducible factor is considered to be one of the primary regulatory pathways involved in hypoxia tolerance (Wang and Semenza 1993). Our screen included HIF1α/sima P-element line, and we found that the loss of sima in the P-element line showed similar phenotype of eclosion under hypoxia as controls. This is consistent with the previous study that showed that sima loss of function affects development under hypoxia (Centanin *et al.* 2005). As our screen is based on the phenotype of hypoxia tolerance, it is reassuring to see that the sima mutant did affect hypoxia tolerance and had low eclosion rates (less than 70%). This explains why we could not detect the role of HIF1α/sima, which is a major hypoxia-sensitive pathway, in our study. We also discovered that that hypoxia tolerance is polygenic as other pathways, such as Wnt, JNK, or Hedgehog, were linked to the candidate genes and played a role in hypoxia tolerance (Table 2). Our future goal is to study the mechanism(s) of hypoxia tolerance as mediated by these genes through genetic epistasis or interaction studies.

Other possibilities may also regulate the interplay of Osa and Wg signaling, such as mutual transcriptional regulation of common target genes (Baig *et al.* 2010). In vertebrates, direct transcriptional regulation of cyclins by SWI/SNF complex (Osa mammalian ortholog) components has been implicated, and mammalian BRG1 (Osa-Brm complex) and β-catenin (the vertebrate ortholog of Armadillo) interact with each other to activate Wnt target genes (Baig *et al.* 2010). Similarly, other mechanism(s) may be responsible for our observed hypoxia-tolerant phenotype. Our observation of the specific role of lqf in larval hemocytes in eclosion under hypoxia may be related to its autophagic function (Csikos *et al.* 2009). During the larval stage, hemocytes play an important role in adult and pupae structural remodeling involving both their phagocytotic (apoptotic cells) as well as their immune function (Holz *et al.* 2003). Furthermore, in our screen, we found the tumor suppressor gene, lethal(3)malignant, which is required for the differentiation of hemocytes (Konrad *et al.* 1994). The P-element line in which this gene was upregulated showed strong eclosion under hypoxia, which reinforces the role of specific genes affecting hemocyte functions and thereby altering hypoxia tolerance (Azad *et al.* 2009).

In summary, the P-element screen is a distinct method for identifying genes that lead to hypoxia tolerance in *Drosophila*. Indeed, by screening 2187 lines, we identified 44 strong hypoxia-tolerant lines (44 genes). The genes found in our screen not only play a role in hypoxia tolerance during development but also help in adult survival one week post eclosion. Of interest, we found that among the 44 lines that seemed hypoxia tolerant, a few genes (*Drp1*,*CG10700*, *CG30169*, *l(3)mbn*, *CG5235*, *polo*, *lqf*, *CG6028*, *sec8*, *Cyclin E*, *Atg1*, *mRpS18B*, and *ci*) were similar to those in our previous work on the hypoxia-adapted adult flies as well on the adapted *Drosophila* larvae (Zhou *et al.* 2007, 2008). This clearly reinforces the potential role of such genes in hypoxia tolerance. Furthermore, in this screen, for the first time we have discovered the distinct role of *osa* and *lqf* genes in hypoxia tolerance by over expressing or knocking down these genes *in vivo* ubiquitously or in specific tissues in *Drosophila*.

## Supplementary Material

Supporting Information
