# Supplementary material for: Identification of Genes Underlying Hypoxia Tolerance in Drosophila by a P-element Screen
Source: G3 (Bethesda). 2012 Oct 1;2(10):1169–78. doi: 10.1534/g3.112.003681 (PMC3464109; doi:10.1534/g3.112.003681)
Supplement: Supporting Information [file supp_2_10_1169__index.html]

Supporting Information 

# Identification of Genes Underlying Hypoxia Tolerance in *Drosophila* by a P-element Screen

## Supporting Information for Azad *et al.*, 2012

**Files in this Data Supplement:**

- Supporting Information - Tables S1 and S2 (PDF, 319 KB)
- Table S1 - Primer sequences of genes tested by real time PCR (PDF, 56 KB)
- Table S2 - Total Genes Screened In Psup P-Element Screen (PDF, 314 KB)
