# Supplementary material for: Identification of Genes Underlying Hypoxia Tolerance in Drosophila by a P-element Screen
Source: G3 (Bethesda). 2012 Oct 1;2(10):1169–78. doi: 10.1534/g3.112.003681 (PMC3464109; doi:10.1534/g3.112.003681)
Supplement: Supporting Information [file supp_2.10.1169_003681SI.pdf]

Table S1 Primer sequences of genes tested by real time PCR

GENE NAME	PRIMER SEQUENCE
Sec8-L	TTGCCATCATAGTGGAGTCG
Sec8-R	CGTTGAGCAACTCTGTTTGG
CG10700-L	ACATTGGGCGAGTTGATAGG
CG10700-R	CCCAAAATCCTGATGATTCC
Osa-L	CATCTCAAAACAGCCACTCG
Osa-R	CTTCCAGGTCTTGCTCTTGG
mRpS18B-L	CAACATTACCCGGAATAGCC
mRpS18B-R	CCGCATTATTTTCTTCAGTCG
l(3)mbn-L	TGTGACAACGACTCCAAAGG
l(3)mbn-R	TAGGAAACAACCGGAACAGG
Atg1-L	CTGAAAATTGCGGATTTTGG
Atg1-R	CGCCATATACATGGGAGAGC
CG5235-L	CGCTATCCCGAGTTTACAGC
CG5235-R	AATTGTGAACTTCCCGAACG
Cpa-L	CGAAGTGTTCAACGATGTGC
Cpa-R	CTTGTTGTATTGGGCGAAGG
Mys45A-L	CACCTCGCTCAAGTTCTTCC
Mys45A-R	TAAGGTCCACCTCGTTCTCG
Iqf-L	GGTTGGCTGCAAAGTAATGG
lqf-R	TTTATCAGCCAGCCTTCAGC
tna-L	AGTTCTATCCGGGCAGTGG
tna-R	GCTGGAAACCTTGATTCTGG
Scrib-L	AATAAGCCAGCTGACGAAGC
Scrib-R	CGGCAGATAACCCAAATAGG
Drp1-L	AGGATGTCTCCGACAAGAGC
Drp1-R	TTCCGTGGTCTCGATATTCC
CG6230-L	GTACCCACTCCCAACAATGG
CG6230-R	TGTCTTCCGATCCTCATTCC
CG6860-L	AATACGATTCGCAGCATTCC
CG6860-R	GAGGCAGGAAACAAATCTCG
Rep2-L	GGGTTAGGCTTTCGACTTCC
Rep2-R	AACGTACGTGCTTCCTTTCG
Bgb-1	TGGCTGTCTGGAGTATGACG
Bgb-2	AACTAACGACGCCACATGC
CG17273-L	ACACGTGCATCTGTCTGACC
CG17273-R	GGAAGTGGTCAAGCTTCTCG

CG8116-L	CACTGTGCATCATCGAGACC
CG8116-R	AATATTACGGATGCGGTTGC
CG8180-L	GCAAAGCGAGACAAAAGAGC
CG8180-R	CAAGGAGCCAAGTTTTCTGC
Manf-L	CGACTTCATCAAGCGTATCG
Manf-R	TAGCTAAGTGGGCGTGTTCC
Ci-L	GCAGCGGCTACTGTTTTACC
Ci-R	CCCGTTGGAATAATTGATGG

Table S2 Total Genes Screened In Psup P-Element Screen

Gene Symbol	Gene Name	Annotation ID
a	arc	CG6741
Act5C	Actin 5C	CG4027
Act57B	Actin 57B	CG10067
Adh	Alcohol dehydrogenase	CG3481
Adhr	Adh-related	CG3484
aop	anterior open	CG3166
Aprt	Adenine phosphoribosyltransferase	CG18315
Argk	Arginine kinase	CG32031
aub	aubergine	CG6137
bib	big brain	CG4722
bic	bicaudal	CG3644
BicC	Bicaudal C	CG4824
br	broad	CG11491
Bsg25A	Blastoderm-specific gene 25A	CG12205
bsk	basket	CG5680
by	blistery	CG9379
cact	cactus	CG5848
Cam	Calmodulin	CG8472
Cf2	Chorion factor 2	CG11924
cg	combgap	CG8367
Cha	Choline acetyltransferase	CG12345
cni	cornichon	CG5855
cup	cup	CG11181
CycB	Cyclin B	CG3510
Hr46	Hormone receptor-like in 46	CG33183
Dip-B	Dipeptidase B	CG9285
Lar	Leukocyte-antigen-related- like	CG10443
dnc	dunce	CG32498
Pka-C3	cAMP-dependent protein kinase 3	CG6117
dpp	decapentaplegic	CG9885
ea	easter	CG4920
eas	easily shocked	CG3525
E(bx)	Enhancer of bithorax	CG32346
ec	echinus	CG2904
EcR	Ecdysone receptor	CG1765
ed	echinoid	CG12676
Ef2b	Elongation factor 2b	CG2238

Eip55E	Eip55E	CG5345
Eip75B	Ecdysone-induced protein 75B	CG8127
Eno	Enolase	CG17654
esc	extra sexcombs	CG14941
exd	extradenticle	CG8933
fas	faint sausage	CG17716
Fas3	Fasciclin 3	CG5803
fj	four-jointed	CG10917
Actn	α actinin	CG4376
flw	flapwing	CG2096
Fmrf	FMRFamide-related	CG2346
for	foraging	CG10033
fy	fuzzy	CG13396
G-iα65A	G protein αi subunit 65A	CG10060
Gβ13F	G protein β-subunit 13F	CG10545
Gl	Glued	CG9206
G-oα47A	G protein oα 47A	CG2204
Got2	Glutamate oxaloacetate transaminase 2	CG4233
Gpdh	Glycerol 3 phosphate dehydrogenase	CG9042
gro	groucho	CG8384
gt	giant	CG7952
her	hermaphrodite	CG4694
Hex-C	Hexokinase C	CG8094
His2A	Histone H2A	-
His3	Histone H3	-
His4	Histone H4	-
hk	hook	CG10653
Hsc70-3	Heat shock protein cognate 3	CG4147
Hsp26	Heat shock protein 26	CG4183
Hsp27	Heat shock protein 27	CG4466
ImpL2	Ecdysone-inducible gene L2	CG15009
inv	invected	CG17835
ix	intersex	CG13201
kay	kayak	CG33956
kel	kelch	CG7210
klar	klarsicht	CG17046
Kr	Kruppel	CG3340
ifc	infertile crescent	CG9078
Sos	Son of sevenless	CG7793

stc	shuttle craft	CG3647
Gli	Gliotactin	CG3903
Ca-α1D	2+ Ca-channel protein α1 subunit D	CG4894
crp	cropped	CG7664
swm	second mitotic wave missing	CG10084
Aats-asp	Aspartyl-tRNA synthetase	CG3821
l(2)gl	lethal (2) giant larvae	CG2671
Pros26	Proteasome 26kD subunit	CG4097
l(3)mbn	lethal (3) malignant blood neoplasm	CG12755
sti	sticky	CG10522
LanA	Laminin A	CG10236
HLHm7	E(spl) region transcript m7	CG8361
mam	mastermind	CG8118
Map205	Microtubule-associated protein 205	CG1483
Men	Malic enzyme	CG10120
mago	mago nashi	CG9401
msl-3	male-specific lethal 3	CG8631
mnd	minidiscs	CG3297
mod(mdg4)	modifier of mdg4	CG32491
mr	morula	CG3060
mus201	mutagen-sensitive 201	CG10890
mus301	mutagen-sensitive 301	CG7972
neur	neuralized	CG11988
ninaE	neither inactivation nor afterpotential E	CG4550
nkd	naked cuticle	CG11614
numb	numb	CG3779
osp	outspread	CG3479
otu	ovarian tumor	CG12743
ovo	ovo	CG6824
pbl	pebble	CG8114
Pc	Polycomb	CG32443
per	period	CG2647
phl	pole hole	CG2845
pk	prickle	CG11084
pnt	pointed	CG17077
polo	polo	CG12306
PpV	Protein phosphatase V	CG12217
pum	pumilio	CG9755
ras	raspberry	CG1799

ref(2)P	refractory to sigma P	CG10360
rt	rotated abdomen	CG6097
sax	saxophone	CG1891
sca	scabrous	CG17579
sd	scalloped	CG8544
sesB	stress-sensitive B	CG16944
sgg	shaggy	CG2621
sha	shavenoid	CG13209
shi	shibire	CG18102
shn	schnurri	CG7734
skd	skuld	CG9936
slgA	sluggish A	CG1417
sm	smooth	CG9218
smo	smoothened	CG11561
stwl	stonewall	CG3836
Sod	Superoxide dismutase	CG11793
spir	spire	CG10076
spz	spatzle	CG6134
sta	stubarista	CG14792
stg	string	CG1395
Su(dx)	Suppressor of deltex	CG4244
su(f)	suppressor of forked	CG17170
su(s)	suppressor of sable	CG6222
sw	short wing	CG18000
CG16778	-	CG16778
tkv	thickveins	CG14026
Egfr	Epidermal growth factor receptor	CG10079
tra2	transformer 2	CG10128
Treh	Trehalase	CG9364
trx	trithorax	CG8651
tsh	teashirt	CG1374
ttk	tramtrack	CG1856
αTub84B	α-Tubulin at 84B	CG1913
tud	tudor	CG9450
snRNA:U2:38ABb	small nuclear RNA U2 at 38ABb	CR32878
vap	vacuolar peduncle	CG9209
vls	valois	CG10728
w	white	CG2759
Yp3	Yolk protein 3	CG11129

uzip	unzipped	CG3533
Zw	Zwischenferment	CG12529
blow	blown fuse	CG1363
Mst84Dc	Male-specific RNA 84Dc	CG17945
Peb	Protein ejaculatory bulb	CG2668
snRNA:U1:82Eb	small nuclear RNA U1 at 82Eb	CR32862
ct	cut	CG11387
Hrb87F	Heterogeneous nuclear ribonucleoprotein at 87F	CG12749
Syt1	Synaptotagmin 1	CG3139
HmgD	High mobility group protein D	CG17950
Ptp4E	Protein tyrosine phosphatase 4E	CG6899
neb	nebbish	CG10718
Klp64D	Kinesin-like protein at 64D	CG10642
Klp68D	Kinesin-like protein at 68D	CG7293
Gap1	GTPase-activating protein 1	CG6721
rhi	rhino	CG10683
Pep	Protein on ecdysone puffs	CG6143
Gα49B	G protein α49B	CG17759
UbcD6	Ubiquitin conjugating enzyme	CG2013
Ten-m	Tenascin major	CG5723
Pk17E	Protein kinase-like 17E	CG7001
Mdr65	Multiple drug resistance 65	CG10181
Gad1	Glutamic acid decarboxylase 1	CG14994
bgcn	benign gonial cell neoplasm	CG30170
Rrp1	Recombination repair protein 1	CG3178
Src42A	Src oncogene at 42A	CG7873
Plc21C	Phospholipase C at 21C	CG4574
rho	rhomboid	CG1004
drk	downstream of receptor kinase	CG6033
N	Notch	CG3936
svr	silver	CG4122
yl	yolkless	CG1372
fru	fruitless	CG14307
Pgd	Phosphogluconate dehydrogenase	CG3724
wapl	wings apart-like	CG3707
fs(1)h	female sterile (1) homeotic	CG2252
mys	myospheroid	CG1560
mdy	midway	CG31991
Takr86C	Tachykinin-like receptor at 86C	CG6515

ci	cubitus interruptus	CG2125
hop	hopscotch	CG1594
Eip78C	Ecdysone-induced protein 78C	CG18023
Gdi	GDP dissociation inhibitor	CG4422
bab1	bric a brac 1	CG9097
enc	encore	CG10847
cdi	center divider	CG6027
scrt	scratch	CG1130
tok	tolkin	CG6863
14-3-3ζ	14-3-3ζ	CG17870
Hnf4	Hepatocyte nuclear factor 4	CG9310
Top1	Topoisomerase 1	CG6146
Sam-S	S-adenosylmethionine Synthetase	CG2674
sno	strawberry notch	CG1903
ewg	erect wing	CG3114
Sox15	Sox box protein 15	CG8404
msl-2	male-specific lethal 2	CG3241
flam	flamenco	-
lola	longitudinals lacking	CG12052
wdn	wings down	CG1454
Pabp2	Pabp2	CG2163
Aats-glupro	Glutamyl-prolyl-tRNA synthetase	CG5394
noc	no ocelli	CG4491
Con	Connectin	CG7503
PpD5	Protein phosphatase D5	CG10138
PpD6	Protein phosphatase D6	CG8822
hbn	homeobrain	CG33152
Cyp4g1	Cytochrome P450-4g1	CG3972
GstD3	Glutathione S transferase D3	CG4381
hdc	headcase	CG15532
Gyc32E	Guanyl cyclase at 32E	CG33114
Sod2	Superoxide dismutase 2 (Mn)	CG8905
ATPsyn-β	ATP synthase-β	CG11154
HmgZ	HMG protein Z	CG17921
Rbp4	RNA-binding protein 4	CG9654
brat	brain tumor	CG10719
pigeon	pigeon	CG10739
CycD	Cyclin D	CG9096
Map60	Microtubule-associated protein 60	CG1825

CycE	Cyclin E	CG3938
Dbi	Diazepam-binding inhibitor	CG8627
RpL18A	Ribosomal protein L18A	CG6510
RpS19a	Ribosomal protein S19a	CG4464
Sdc	Syndecan	CG10497
TfIIS	RNA polymerase II elongation factor	CG3710
mtrm	matrimony	CG18543
kermit	kermit	CG11546
CCS	CCS	CG17753
sbb	scribbler	CG5580
l(2)08717	lethal (2) 08717	CG15095
Gug	Grunge	CG6964
l(3)05822	lethal (3) 05822	CG7129
pelo	pelota	CG3959
blw	bellwether	CG3612
jar	jaguar	CG5695
Sema-2a	Sema-2a	CG4700
Pbprp5	Pheromone-binding protein- related protein 5	CG6641
Ssdp	Sequence-specific single- stranded DNA-binding protein	CG7187
Iswi	Imitation SWI	CG8625
Mad	Mothers against dpp	CG12399
Mef2	Myocyte enhancer factor 2	CG1429
msi	musashi	CG5099
Mvl	Malvolio	CG3671
pav	pavarotti	CG1258
rost	rolling stone	CG9552
rpr	reaper	CG4319
ctp	cut up	CG6998
Dp	DP transcription factor	CG4654
E2f	E2F transcription factor	CG6376
nmo	nemo	CG7892
oaf	out at first	CG9884
Ret	Ret oncogene	CG14396
tRNA:H:56E	transfer RNA:his:56E	CR30233
Aldh	Aldehyde dehydrogenase	CG3752
CalpA	Calpain-A	CG7563
Trl	Trithorax-like	CG33261
l(2)dtl	lethal-(2)-denticleless	CG11295
l(3)82Fd	l(3)82Fd	CG32464

nuf	nuclear fallout	CG33991
pnut	peanut	CG8705
shot	short stop	CG18076
Arf51F	ADP ribosylation factor 51F	CG8156
Bgb	Big brother	CG7959
CG5210	-	CG5210
cnn	centrosomin	CG4832
FK506-bp2	FK506-binding protein 2	CG11001
Gycβ100B	Guanylyl cyclase β-subunit at 100B	CG1470
InR	Insulin-like receptor	CG18402
Pak	PAK-kinase	CG10295
Rab2	Rab-protein 2	CG3269
Rab5	Rab-protein 5	CG3664
Rac2	Rac2	CG8556
Rel	Relish	CG11992
Rh5	Rhodopsin 5	CG5279
Rho1	Rho1	CG8416
CG4278	-	CG4278
bif	bifocal	CG1822
bnl	branchless	CG4608
fax	failed axon connections	CG4609
gcm	glial cells missing	CG12245
Cyp4e2	Cytochrome P450-4e2	CG2060
His3.3A	Histone H3.3A	CG5825
Cyp9b2	Cytochrome P450-9b2	CG4486
eIF-4E	Eukaryotic initiation factor 4E	CG4035
Nap1	Nucleosome assembly protein 1	CG5330
Rala	Ras-related protein	CG2849
RpL22	Ribosomal protein L22	CG7434
Shc	SHC-adaptor protein	CG3715
chrw	chrowded	CG3870
jumu	jumeau	CG4029
nAcRα-7E	nicotinic Acetylcholine Receptor alpha 7E	CG2302
olf186-M	olf186-M	CG14489
sima	similar	CG7951
α-Est10	α-Esterase-10	CG1131
toc	toucan	CG9660
BEAF-32	Boundary element-associated factor of 32kD	CG10159
Dot	Dorothy	CG2788

Dref	DNA replication-related element factor	CG5838
Cyp6a17	Cyp6a17	CG10241
Mpk2	Mpk2	CG5475
NetB	Netrin-B	CG10521
P5cr	Pyrroline 5-carboyxlate reductase	CG6009
Slh	SLY-1 homologous	CG3539
Trip1	Trip1	CG8882
apt	apontic	CG5393
ast	asteroid	CG4426
bl	bancal	CG13425
crq	croquemort	CG4280
hrg	hiiragi	CG9854
l(2)55BCa	lethal (2) 55BCa	-
l(2)55Da	lethal (2) 55Da	-
l(2)55Db	lethal (2) 55Db	-
l(3)100CDa	lethal (3) 100CDa	-
l(3)61Da	lethal (3) 61Da	-
l(3)73CFa	lethal (3) 73CFa	-
l(3)85Ak	lethal (3) 85Ak	-
l(3)87Em	lethal (3) 87Em	-
lama	lamina ancestor	CG10645
mael	maelstrom	CG11254
mod(r)	modifier of rudimentary	CG17828
vri	vrille	CG14029
Cdk4	Cyclin-dependent kinase 4	CG5072
Pdp1	PAR-domain protein 1	CG17888
Rab4	Rab-protein 4	CG4921
Ubp64E	Ubiquitin-specific protease 64E	CG5486
fz2	frizzled 2	CG9739
Pino	Pinocchio	CG4710
cag	cag	CG12346
Lk6	Lk6	CG17342
Ser8	Ser8	CG4812
SamDC	S-adenosylmethionine decarboxylase	CG5029
Cbl	Cbl	CG7037
ATPCL	ATP citrate lyase	CG8322
ttv	tout-velu	CG10117
stet	stem cell tumor	CG33166
sfl	sulfateless	CG8339

ppa	partner of paired	CG9952
lush	lush	CG8807
loco	locomotion defects	CG5248
lig	lingerer	CG8715
gk	geko	CG13695
dbe	dribble	CG4258
dve	defective proventriculus	CG5799
crol	crooked legs	CG14938
TppII	tripeptidyl-peptidase II	CG3991
Tim17b2	Translocase inner membrane 17	CG15257
Dredd	Death related ced-3/Nedd2- like protein	CG7486
Pdk1	Phosphoinositide-dependent kinase 1	CG1210
JIL-1	JIL-1	CG6297
Fak56D	Focal Adhesion Kinase	CG10023
Elf	Ef1α-like factor	CG6382
E23	Early gene at 23	CG3327
CtBP	C-terminal Binding Protein	CG7583
CLIP-190	Cytoplasmic linker protein 190	CG5020
ade5	ade5	CG3989
kraken	kraken	CG3943
Pkn	Protein kinase related to protein kinase N	CG2049
KrT95D	Krueppel target at 95D	CG5405
Atet	ABC transporter expressed in trachea	CG2969
Alas	Aminolevulinate synthase	CG3017
RpL3	Ribosomal protein L3	CG4863
Crg-1	Circadianly Regulated Gene	CG32788
chb	chromosome bows	CG32435
sdk	sidekick	CG5227
l(2)k16918	lethal (2) k16918	CG3920
l(2)k14710	lethal (2) k14710	CG8325
ytr	yantar	CG18426
Pdsw	Pdsw	CG8844
Rpp30	RNaseP protein p30	CG11606
CG1910	-	CG1910
Pur-α	Purine-rich binding protein-α	CG1507
D19B	D19B	CG10270
Adk1	Adenylate kinase-1	CG17146
CycH	Cyclin H	CG7405
qkr58E-2	quaking related 58E-2	CG5821

CRMP	Collapsin Response Mediator Protein	CG1411
gek	genghis khan	CG4012
fray	frayed	CG7693
dimm	dimmed	CG8667
caps	capricious	CG11282
a6	a6	CG3771
Mnt	Mnt	CG13316
B4	B4	CG9239
CG14814	-	CG14814
CG3857	-	CG3857
CG17778	-	CG17778
arg	arginase	CG18104
CG17896	-	CG17896
CG4406	-	CG4406
san	separation anxiety	CG12352
ird5	immune response deficient 5	CG4201
Fim	Fimbrin	CG8649
chico	chico	CG5686
brk	brinker	CG9653
trio	trio	CG18214
Sir2	Sir2	CG5216
Mekk1	Mekk1	CG7717
E2f2	E2F transcription factor 2	CG1071
Dlc90F	Dynein light chain 90F	CG12363
fws	four way stop	CG6549
Flo-2	flotillin 2	CG32593
Flo	flotillin	CG8200
DIP2	DISCO Interacting Protein 2	CG7020
Crk	Crk	CG1587
p38b	p38b	CG7393
NTPase	NTPase	CG3059
GluClα	GluClα	CG7535
CG3719	-	CG3719
CG3777	-	CG3777
Ant2	Adenine nucleotide translocase 2	CG1683
Rab27	Rab27	CG14791
Scgδ	Sarcoglycan δ	CG14808
slv	saliva	CG8717
fidipidine	fidipidine	CG7773

CG16989	-	CG16989
CG4313	-	CG4313
skpA	skpA	CG16983
Hmt4-20	Histone methyltransferase 4-20	CG13363
CG32812	-	CG32812
CycK	Cyclin K	CG15218
CG3558	-	CG3558
CG3164	-	CG3164
CG11163	-	CG11163
plexB	plexin B	CG17245
TBPH	TBPH	CG10327
SNF4Aγ	SNF4/AMP-activated protein kinase gamma subunit	CG17299
Mgstl	Microsomal glutathione S- transferase-like	CG1742
CG2652	-	CG2652
Inos	Inos	CG11143
Mipp2	Multiple inositol polyphosphate phosphatase 2	CG4317
KP78a	KP78a	CG6715
CDC45L	CDC45L	CG3658
CBP	sarcoplasmic calcium-binding protein	CG1435
tna	tonalli	CG7958
siz	schizo	CG32434
clumsy	clumsy	CG8681
eIF5B	eIF5B	CG10840
Tom	Twin of m4	CG5185
Taf10b	TBP-associated factor 10b	CG3069
Sep5	Sep5	CG2916
RhoGAPp190	RhoGAPp190	CG32555
Nc	Nedd2-like caspase	CG8091
Hus1-like	Hus1-like	CG2525
Hsc70Cb	Hsc70Cb	CG6603
Drp1	Dynamin related protein 1	CG3210
CG8677	-	CG8677
CG9418	-	CG9418
Fie	Fire exit	CG1079
Ady43A	Ady43A	CG1851
α-Man-IIb	α-Man-IIb	CG4606
Taz	Tafazzin	CG8766
l(1)G0007	lethal (1) G0007	CG32604
mRpL18	mitochondrial ribosomal protein L18	CG12373

Trap1	Trap1	CG3152
CG11403	-	CG11403
CG17322	-	CG17322
Manf	Mesencephalic astrocyte- derived neurotrophic factor	CG7013
inx7	innexin 7	CG2977
Gmap	Golgi microtubule-associated protein	CG33206
Rip11	Rab11 interacting protein	CG6606
fz4	frizzled 4	CG4626
fz3	frizzled 3	CG16785
Tim10	Translocase of inner membrane 10	CG9878
wdb	widerborst	CG5643
CG17273	-	CG17273
CG11970	-	CG11970
EDTP	Egg-derived tyrosine phosphatase	CG6542
CG7115	-	CG7115
CG3909	-	CG3909
Osi6	Osiris 6	CG1151
CG11092	-	CG11092
β4GalNAcTA	β4GalNAcTA	CG8536
CG1927	-	CG1927
CG10863	-	CG10863
pgant3	polypeptide GalNAc transferase 3	CG4445
CG8108	-	CG8108
Nep2	Neprilysin 2	CG9761
CG3523	-	CG3523
CG5815	-	CG5815
CG6230	-	CG6230
CG10249	-	CG10249
cindr	CIN85 and CD2AP orthologue	CG31012
Vps4	Vacuolar protein sorting 4	CG6842
morgue	modifier of rpr and grim, ubiquitously expressed	CG15437
Acf1	ATP-dependent chromatin assembly factor large subunit	CG1966
Pfrx	6-phosphofructo-2-kinase	CG3400
glob1	globin 1	CG9734
vih	vihar	CG10682
ppl	pumpless	CG7758
l(1)G0334	lethal (1) G0334	CG7010
l(1)G0320	lethal (1) G0320	CG32701
l(1)G0148	lethal (1) G0148	CG32742

kirre	kin of irre	CG3653
jbug	jitterbug	CG30092
CG17834	-	CG17834
Tob	Tob	CG9214
Taf10	TBP-associated factor 10	CG2859
Drep-4	DNA fragmentation factor- related protein 4	CG9414
Drep-2	DNA fragmentation factor- related protein 2	CG1975
Mst33A	Mst33A	CG6541
Kr-h2	Kruppel homolog 2	CG9159
Kr-h1	Kruppel homolog 1	CG18783
Kap3	Kinesin associated protein 3	CG11759
JhI-26	Juvenile hormone-inducible protein 26	CG3767
JhI-21	JhI-21	CG12317
JhI-1	Juvenile hormone-inducible protein 1	CG3298
CG17841	-	CG17841
CG6424	-	CG6424
CG3530	-	CG3530
CG12182	-	CG12182
CG3793	-	CG3793
cenG1A	centaurin gamma 1A	CG31811
CG18507	-	CG18507
Atf3	Activating transcription factor 3	CG11405
sut3	sugar transporter 3	CG17976
robl22E	robl22E	CG10838
phtf	phtf	CG3268
lqf	liquid facets	CG8532
VhaM9.7-b	+ Vacuolar H ATPase subunit M9.7-b	CG7625
Rpn2	Rpn2	CG11888
Nhe3	Na+/H+ hydrogen exchanger 3	CG11328
Mst85C	Mst85C	CG11993
Fmr1	Fmr1	CG6203
CG5033	-	CG5033
CG15262	-	CG15262
CG18477	-	CG18477
CG31817	-	CG31817
CG3473	-	CG3473
CG14478	-	CG14478
γCop	γ-coatomer protein	CG1528
δCOP	δ-coatomer protein	CG14813

trbl	tribbles	CG5408
tio	tiptop	CG12630
Spn3	Serine protease inhibitor 3	CG9334
seq	sequoia	CG32904
lack	lethal with a checkpoint kinase	CG4943
ced-6	ced-6	CG11804
Sras	severas	CG4852
REG	REG	CG1591
Men-b	Malic enzyme b	CG5889
CHES-1-like	Checkpoint suppressor homologue	CG12690
Tsp42Ea	Tetraspanin 42Ea	CG18817
CG14635	-	CG14635
CG14632	-	CG14632
CG3603	-	CG3603
CG2941	-	CG2941
CG7024	-	CG7024
CG2861	-	CG2861
CG4068	-	CG4068
yu	yu	CG3249
CG12730	-	CG12730
CG12239	-	CG12239
CG3011	-	CG3011
Grip	Glutamate receptor binding protein	CG14447
CG5941	-	CG5941
CG5937	-	CG5937
Nep1	Neprilysin 1	CG5905
CG3823	-	CG3823
CG15893	-	-
CG14438	-	CG14438
CG9650	-	CG9650
CG15035	-	CG15035
Upf2	Upf2	CG2253
CG1575	-	CG1575
cyr	cypher	CG15335
CG2116	-	CG2116
sni	sniffer	CG10964
CG1440	-	CG1440
CG11284	-	CG11284
CG11294	-	CG11294

CG2004	-	CG2004
CG12661	-	CG12661
CG10962	-	CG10962
CG10970	-	CG10970
fend	forked end	CG12664
dalao	dalao	CG7055
RpS28b	Ribosomal protein S28b	CG2998
Gga	Golgi-localized, γ-adaptin ear containing, ARF binding protein	CG3002
CG15249	-	CG15249
CG2908	-	-
CG15306	-	CG15306
CG12641	-	CG12641
CG1628	-	CG1628
CG9806	-	CG9806
CG1826	-	CG1826
CG15207	-	CG15207
CG11203	-	CG11203
CG1737	-	CG1737
CG1572	-	CG1572
CG11696	-	CG11696
rho-4	rhomboid-4	CG1697
Amun	Amun	CG2446
CG15221	-	CG15221
p24-1	p24-related-1	CG1967
CG10353	-	CG10353
CG1806	-	CG1806
Usp7	ubiquitin-specific protease 7	CG1490
CG15730	-	CG15730
CG2556	-	CG2556
CG11138	-	CG11138
CG4395	-	CG4395
CG15717	-	CG15717
NFAT	NFAT homolog	CG11172
Clic	Chloride intracellular channel	CG10997
CG13403	-	CG13403
CG9413	-	CG9413
CG14411	-	CG14411
CG14407	-	CG14407
CG9512	-	CG9512

CG9030	-	CG9030
hiw	highwire	CG32592
CG5548	-	CG5548
Lsd-2	Lipid storage droplet-2	CG9057
CG5599	-	CG5599
CG9203	-	CG9203
CG6324	-	CG6324
CG7872	-	CG7872
CG8097	-	CG8097
CG9281	-	CG9281
CG15601	-	CG15601
CG8191	-	CG8191
PGRP-LE	Peptidoglycan recognition protein LE	CG8995
CG8509	-	CG8509
dpr18	dpr18	CG14948
CG3560	-	CG3560
CG9921	-	CG9921
CG9906	-	CG9906
CG9784	-	CG9784
CG13012	-	CG13012
mRpL22	mitochondrial ribosomal protein L22	CG4742
CG4768	-	CG4768
CG4949	-	CG4949
CG4991	-	CG4991
CG8926	-	-
IntS2	Integrator 2	CG8211
CG8188	-	CG8188
CG6788	-	CG6788
CG6106	-	CG6106
Ggt-1	γ-glutamyl transpeptidase	CG6461
CCKLR-17D3	CCK-like receptor at 17D3	CG32540
CG6891	-	CG6891
CG7101	-	CG7101
CG7349	-	CG7349
CG7453	-	CG7453
CG8051	-	CG8051
kek5	kekkon5	CG12199
Tyler	Tyler	CG14208
MKP-4	MAPK Phosphatase 4	CG14211

Rcd-1	Required for cell differentiation 1 ortholog	CG14213
Arp11	Arp11	CG12235
CG14232	-	CG14232
Alr	Augmenter of liver regeneration	CG12534
CG12703	-	CG12703
CG9577	-	CG9577
CG1631	-	CG1631
CG1702	-	CG1702
Ntf-2	Nuclear transport factor-2	CG1740
CG1518	-	CG1518
bves	bves	CG32513
CG11566	-	CG11566
CG12576	-	CG12576
CG17601	-	CG17601
CG11454	-	CG11454
CG3645	-	CG3645
CG3345	-	CG3345
CG3625	-	CG3625
CG11885	-	CG11885
CG13692	-	CG13692
CG4133	-	CG4133
CG3862	-	CG3862
IA-2	IA-2 ortholog	CG31795
CG4764	-	CG4764
CG4896	-	CG4896
CG17646	-	CG17646
CG15358	-	CG15358
CG7337	-	CG7337
Npc2a	Niemann-Pick type C-2a	CG7291
CG4238	-	CG4238
CG4259	-	CG4259
CG11723	-	CG11723
CG10874	-	CG10874
CG10880	-	CG10880
papi	papi	CG7082
CG9866	-	CG9866
VGlut	Vesicular glutamate transporter	CG9887
CG9886	-	CG9886
CG15393	-	CG15393

CG3528	-	CG3528
CG3515	-	CG3515
Cyp309a1	Cyp309a1	CG9964
CG9883	-	CG9883
CG15395	-	CG15395
CG31689	-	CG31689
CG9894	-	CG9894
Trn-SR	Transportin-Serine/Arginine rich	CG2848
CG3077	-	CG3077
aph-1	anterior pharynx defective 1	CG2855
CG2862	-	CG2862
CG15399	-	CG15399
daw	dawdle	CG16987
CG15400	-	CG15400
Duox	Dual oxidase	CG3131
Cpr23B	Cuticular protein 23B	CG2973
CG2975	-	CG2975
CG18558	-	CG18558
CG18557	-	CG18557
CG3117	-	CG3117
CG3104	-	CG3104
CG2991	-	CG2991
CG17224	-	CG17224
CG17259	-	CG17259
CG3347	-	CG3347
CG2818	-	CG2818
Art2	Arginine methyltransferase 2	CG3675
Elp3	Elongator complex protein 3	CG15433
CG15439	-	CG15439
CG15435	-	CG15435
CG11929	-	CG11929
CG3251	-	CG3251
Taf12L	TBP-associated factor 30kD subunit α-2	CG15632
CG15629	-	CG15629
CG3225	-	CG3225
CG3008	-	CG3008
CG15625	-	CG15625
CG3036	-	CG3036
Marcal1	Marcal1	CG3753

CG14043	-	CG14043
CG31650	-	CG31650
pgant5	polypeptide GalNAc transferase 5	CG31651
CG7277	-	CG7277
Cyp6a16Ψ	Cyp6a16Ψ	CR7249
CG14005	-	CG14005
CG11034	-	CG11034
Tsp26A	Tetraspanin 26A	CG9093
CG9107	-	CG9107
CG9109	-	CG9109
CG12393	-	CG12393
CG9135	-	CG9135
CG9140	-	CG9140
CG13993	-	CG13993
CG9175	-	CG9175
CG9486	-	CG9486
retm	real-time	CG9528
CG9531	-	CG9531
DLP	Daxx-like protein	CG9537
CG13771	-	CG13771
CG11327	-	CG11327
Tsp	Thrombospondin	CG11326
CG17377	-	CG17377
Rat1	-	CG10354
CG18304	-	CG18304
CG10158	-	CG10158
CG4495	-	CG4495
CG4496	-	CG4496
CG4502	-	CG4502
CG13786	-	CG13786
CG7164	-	CG7164
CG7149	-	CG7149
CG7102	-	CG7102
CG7231	-	CG7231
pes	peste	CG7228
CG7227	-	CG7227
CG7224	-	CG7224
CG12560	-	CG12560
CG12375	-	CG12375

CG8552	-	CG8552
CG8498	-	CG8498
CG8455	-	CG8455
CG7806	-	CG7806
CG17292	-	CG17292
CG13088	-	CG13088
Dh31	Diuretic hormone 31	CG13094
CG13096	-	CG13096
CG13089	-	CG13089
C1GalTA	Core 1 Galactosyltransferase A	CG9520
CG31886	-	CG31886
CG9525	-	CG9525
CG3769	-	CG3769
CG33298	-	CG33298
Oatp30B	Organic anion transporting polypeptide 30B	CG3811
CG3838	-	CG3838
CG4382	-	CG4382
GlcAT-S	GlcAT-S	CG3881
IP3K1	Inositol 1,4,5-triphosphate kinase 1	CG4026
nAcRα-30D	nicotinic Acetylcholine Receptor α 30D	CG4128
CG5731	-	CG5731
CG5708	-	CG5708
CG5694	-	CG5694
eEF1δ	eEF1δ	CG4912
CG5390	-	CG5390
CG4968	-	CG4968
CG5381	-	CG5381
CG4995	-	CG4995
GATAd	GATAd	CG5034
Sps2	Selenophosphate synthetase 2	CG5025
CG6232	-	CG6232
CG6700	-	CG6700
CG7309	-	CG7309
Samuel	SAM-motif ubiquitously expressed punctatedly localized protein	CG31868
Csl4	Csl4	CG6249
CG6287	-	CG6287
CG6509	-	CG6509
CG17745	-	-
Tom70	Translocase of outer membrane 70	CG6756

CG6785	-	CG6785
CG6770	-	CG6770
RpL7-like	Ribosomal protein L7-like	CG5317
CG14946	-	CG14946
rho-6	rhomboid-6	CG17212
crok	crooked	CG17218
atilla	atilla	CG6579
CG5446	-	CG5446
Oatp33Ea	Organic anion transporting polypeptide 33Ea	CG5427
CG5421	-	CG5421
Ref2	RNA and export factor binding protein 2	CG17031
CG5525	-	CG5525
MRP	Multidrug-Resistance like Protein 1	CG6214
Edem2	Edem2	CG5682
CG5945	-	CG5945
CG16820	-	CG16820
Uvrag	UV-resistance associated gene	CG6116
CG9306	-	CG9306
Ance-3	Ance-3	CG17988
Tpr2	Tetratricopeptide repeat protein 2	CG4599
CG5953	-	CG5953
CG5968	-	CG5968
CG17928	-	CG17928
CG6012	-	CG6012
CG6304	-	CG6304
beat-IIIc	beat-IIIc	CG15138
Lrch	Leucine-rich-repeats and calponin homology domain protein	CG6860
CG15141	-	CG15141
CG5050	-	CG5050
Sgt	small glutamine-rich tetratricopeptide containing protein	CG5094
CG15142	-	CG15142
CG6412	-	CG6412
elfless	elfless	CG15150
Ntf-2r	Nuclear transport factor-2- related	CG10174
CG15161	-	CG15161
CG10336	-	CG10336
CG17323	-	CG17323
ssp3	short spindle 3	CG18397
Nedd8	Nedd8	CG10679

CG10641	-	CG10641
CG15172	-	CG15172
CG10700	-	CG10700
CG17564	-	CG17564
CG10194	-	CG10194
CG10132	-	CG10132
CG10337	-	CG10337
CG13079	-	CG13079
Hakai	Hakai	CG10263
CG10462	-	CG10462
CdGAPr	CdGAPr	CG10538
CG13962	-	CG13962
CG10659	-	CG10659
nesd	nessun dorma	CG10722
mRpS18B	mitochondrial ribosomal protein S18B	CG10757
Arc-p34	Arc-p34	CG10954
CG2614	-	CG2614
CG9323	-	CG9323
CG9328	-	CG9328
CheB38c	Chemosensory protein B 38c	CG14405
CG9331	-	CG9331
CG9336	-	CG9336
CG9338	-	CG9338
CG14401	-	CG14401
sky	skywalker	CG9339
RPA2	Replication protein A2	CG9273
CG9259	-	CG9259
CG12050	-	CG12050
CG9257	-	CG9257
CG9253	-	CG9253
Mcm10	Sensitized chromosome inheritance modifier 19	CG9241
CG8678	-	CG8678
CG8671	-	CG8671
Mio	Mlx interactor	CG18362
Lamp1	Lamp1	CG3305
CG2201	-	CG2201
CG2225	-	CG2225
CG1416	-	CG1416
CG11629	-	CG11629

CG1421	-	CG1421
ttm3	tiny tim 3	CG6691
CG10834	-	CG10834
CG3651	-	CG3651
CG1832	-	CG1832
CG3635	-	CG3635
CG3262	-	CG3262
Tif-IA	Tif-IA	CG3278
CG17486	-	CG17486
d4	d4	CG2682
TpnC4	Troponin C isoform 4	CG12408
l(2)NC136	lethal (2) NC136	CG8426
scaf	scarface	CG11066
CG7791	-	CG7791
gp210	gp210	CG7897
Pngl	PNGase-like	CG7865
CG14591	-	CG14591
Ars2	-	CG7843
Pld	Phospholipase D	CG12110
CG9410	-	CG9410
CG3194	-	CG3194
CG3271	-	CG3271
CG17266	-	CG17266
CG3409	-	CG3409
ZIP1	Zinc/iron regulated transporter -related protein 1	CG9428
CG17002	-	CG17002
Tsp42Ef	Tetraspanin 42Ef	CG12845
Tsp42El	Tetraspanin 42El	CG12840
Gadd45	Gadd45	CG11086
Dscam	Down syndrome cell adhesion molecule	CG17800
CG11107	-	CG11107
CG1600	-	CG1600
CG1360	-	CG1360
CG1358	-	CG1358
CG1399	-	CG1399
CG1882	-	CG1882
CG12822	-	CG12822
Kdm4A	Histone demethylase 4A	CG15835
CG8728	-	CG8728

CG14764	-	CG14764
CG2915	-	CG2915
CG8726	-	CG8726
CG12769	-	CG12769
CG11210	-	CG11210
Cul-4	Cullin-4	CG8711
CG18316	-	CG18316
Nup50	Nucleoporin 50	CG2158
CG8708	-	CG8708
CG14757	-	CG14757
CG8701	-	CG8701
Cyp6a14	Cyp6a14	CG8687
rgr	regular	CG8643
CG8642	-	CG8642
PGRP-SC1b	PGRP-SC1b	CG8577
CG8272	-	CG8272
tsu	tsunagi	CG8781
Mys45A	Mystery 45A	CG8070
CG8026	-	CG8026
CG2063	-	CG2063
CG1968	-	CG1968
Myd88	Myd88	CG2078
CG1888	-	CG1888
Updo	Updo	CG1818
CG1902	-	CG1902
Mmp2	Matrix metalloproteinase 2	CG1794
hebe	hebe	CG1623
CG1663	-	CG1663
CG15863	-	CG15863
CG1418	-	CG1418
CG12128	-	CG12128
oys	oysgedart	CG18445
CG1371	-	CG1371
CG12214	-	CG12214
CAP	CAP	CG18408
Obp46a	Odorant-binding protein 46a	CG12905
Prx2540-2	Peroxiredoxin 2540-2	CG11765
CG11825	-	CG11825
CG12896	-	CG12896

CG12935	-	CG12935
mms4	-	CG12936
CG7712	-	CG7712
CG12384	-	CG12384
CG13204	-	CG13204
CG9027	-	CG9027
CG7777	-	CG7777
tou	toutatis	CG10897
CG9003	-	CG9003
S2P	site-2 protease	CG8988
CG8979	-	CG8979
CG18343	-	CG18343
Prp8	pre-mRNA processing factor 8	CG8877
CG18342	-	-
CG13170	-	CG13170
CG8830	-	CG8830
CG8545	-	CG8545
CG8550	-	CG8550
CG8771	-	CG8771
CG13322	-	CG13322
CG3884	-	CG3884
CG4627	-	CG4627
CG4630	-	CG4630
CG4646	-	CG4646
pex13	peroxin 13	CG4663
fsd	fates-shifted	CG12765
CG4670	-	CG4670
CG4676	-	CG4676
CG4679	-	CG4679
CG4714	-	CG4714
CG17062	-	-
mars	mars	CG17064
mip120	Myb-interacting protein 120	CG6061
CG6145	-	CG6145
CG6701	-	CG6701
RpS23	Ribosomal protein S23	CG8415
CG8468	-	CG8468
CG17386	-	CG17386
CG10202	-	CG10202

Ciao1	Ciao1	CG12797
Cyp6a20	Cyp6a20	CG10245
Cyp317a1	Cyp317a1	CG17453
CG10253	-	CG10253
Lap1	Lap1	CG10255
CG12424	-	CG12424
CG8089	-	CG8089
CG8079	-	CG8079
CG8152	-	CG8152
CG8249	-	CG8249
tun	tungus	CG8253
bdg	bedraggled	CG8291
CG8315	-	CG8315
CG8320	-	CG8320
CG8370	-	CG8370
CG8386	-	CG8386
Asph	Aspartyl β-hydroxylase	CG8421
CG10731	-	CG10731
CG10734	-	CG10734
clu	clueless	CG8443
CG8446	-	CG8446
mrj	mrj	CG8448
CG15704	-	CG15704
CG15711	-	CG15711
CG8306	-	CG8306
CG5065	-	CG5065
CG4802	-	CG4802
CG14479	-	-
CG10933	-	CG10933
Nup75	Nucleoporin 75	CG5733
CG5721	-	CG5721
CG14505	-	CG14505
CG5224	-	CG5224
CG10927	-	CG10927
sec6	sec6	CG5341
CG12613	-	-
Cyp12b2	Cyp12b2	CG15077
CG15083	-	CG15083
Jheh2	Juvenile hormone epoxide hydrolase 2	CG15102

Jheh3	Juvenile hormone epoxide hydrolase 3	CG15106
sano	serrano	CG12758
CG11961	-	CG11961
CG7744	-	CG7744
CG11208	-	CG11208
CG10444	-	CG10444
MED8	Mediator complex subunit 8	CG13867
CG13423	-	CG13423
CG13430	-	CG13430
CG18065	-	CG18065
Mgat1	UDP-GlcNAc:a-3-D- mannoside-β-1,2-N-acetylglucosaminyltransferase I	CG13431
Nnf1a	Nnf1a	CG13434
CG9945	-	CG9945
CG3295	-	CG3295
cpa	capping protein alpha	CG10540
CG15653	-	CG15653
CG10527	-	CG10527
CG15658	-	CG15658
CG10795	-	CG10795
CG10433	-	CG10433
CG15673	-	CG15673
CG10321	-	CG10321
CG10082	-	CG10082
pirk	poor Imd response upon knock-in	CG15678
CG11269	-	CG11269
CG3624	-	CG3624
mRpS29	mitochondrial ribosomal protein S29	CG3633
rad50	rad50	CG6339
CG11362	-	CG11362
CG3927	-	CG3927
CG4294	-	CG4294
CG2852	-	CG2852
CG13511	-	CG13511
RYBP	Ring and YY1 Binding Protein	CG12190
CG3499	-	CG3499
asrij	asrij	CG13533
MED23	Mediator complex subunit 23	CG3695
CG3700	-	CG3700
nahoda	nahoda	CG12781

CG3800	-	CG3800
CG9849	-	CG9849
CG3831	-	CG3831
CG9896	-	CG9896
CG9890	-	CG9890
RpL37b	Ribosomal protein L37b	CG9873
CG12782	-	CG12782
CG3124	-	CG3124
CG9815	-	CG9815
wmd	wing morphogenesis defect	CG3957
CG4019	-	CG4019
CG5431	-	CG5431
CG5554	-	CG5554
CG5569	-	CG5569
CG3735	-	CG3735
CG3860	-	CG3860
MAN1	MAN1	CG3167
CG3209	-	CG3209
CG13577	-	CG13577
CG3394	-	CG3394
slik	Sterile20-like kinase	CG4527
CG15873	-	CG15873
CG4612	-	CG4612
Ir60e	Ionotropic receptor 60e	CG13592
CG4692	-	CG4692
Mmp1	Matrix metalloproteinase 1	CG4859
CG16896	-	CG16896
Ance-5	Ance-5	CG10142
Tina-1	Troponin C-akin-1	CG2803
CG12851	-	CG12851
CG2765	-	CG2765
Vdup1	Vitamin D3 up-regulated protein 1	CG7047
CG13876	-	CG13876
CG16940	-	CG16940
Tudor-SN	Tudor-SN	CG7008
mRpL17	mitochondrial ribosomal protein L17	CG13880
CG17180	-	CG17180
Ppm1	Ppm1	CG12169
Gale	UDP-galactose 4'-epimerase	CG12030

CG3344	-	CG3344
CG13895	-	CG13895
CG13907	-	CG13907
CG9094	-	-
CG12091	-	CG12091
CG13928	-	CG13928
CG8001	-	CG8001
CG13933	-	CG13933
CG9004	-	CG9004
CG14962	-	CG14962
Girdin	Girdin	CG12734
CG14965	-	CG14965
RpL28	Ribosomal protein L28	CG12740
CG17737	-	CG17737
CG12078	-	CG12078
CG14969	-	CG14969
CG12006	-	CG12006
CG12605	-	CG12605
Fit1	Fermitin 1	CG14991
CG14997	-	CG14997
CG1316	-	CG1316
Cip4	Cip4	CG15015
mRpS6	mitochondrial ribosomal protein S6	CG15016
CG15019	-	CG15019
DOR	-	CG11347
CG7465	-	CG7465
CG15876	-	CG15876
Gef64C	Guanine nucleotide exchange factor GEF64C	CG32239
CG10672	-	CG10672
CG4769	-	CG4769
blanks	blanks	CG10630
Eaf6	Eaf6	CG12756
Blimp-1	Blimp-1	CG5249
Ppat-Dpck	Bifunctional Phosphopantetheine adenylyltransferase - Dephospho-CoA kinase	CG10575
Pole2	Pole2	CG10489
Jon65Ai	Jonah 65Ai	CG10475
CG13295	-	CG13295
CG10107	-	CG10107
CG9953	-	CG9953

Cpr65Ec	Cuticular protein 65Ec	CG8634
CG8596	-	CG8596
sec63	sec63	CG8583
CG7506	-	CG7506
Srp9	Signal recognition particle protein 9	CG8268
Atg18	Autophagy-specific gene 18	CG7986
exo70	exo70	CG7127
PGRP-LF	Peptidoglycan recognition protein LF	CG4437
UGP	UGP	CG4347
CG4452	-	CG4452
CG4080	-	CG4080
CG3967	-	CG3967
Nf-YA	Nuclear factor Y-box A	CG3891
phol	pleiohomeotic like	CG3445
CG3552	-	CG3552
path	pathetic	CG3424
Ir67a	Ionotropic receptor 67a	CG12525
CG6767	-	CG6767
CG8177	-	CG8177
CG6685	-	CG6685
CG8003	-	CG8003
CG6418	-	CG6418
CG6272	-	CG6272
CG14143	-	CG14143
Plod	procollagen lysyl hydroxylase	CG6199
CG6175	-	CG6175
CG11658	-	CG11658
RpL10Ab	Ribosomal protein L10Ab	CG7283
CG6928	-	CG6928
ssp	sunspot	CG17153
Tsf2	Transferrin 2	CG10620
CG14115	-	CG14115
CG10133	-	CG10133
CG10116	-	CG10116
CG3919	-	CG3919
Best4	Bestrophin 4	CG7259
CG7841	-	CG7841
CG7372	-	CG7372
GXIVsPLA2	GXIVsPLA2	CG17035

CG5235	-	CG5235
CG5151	-	CG5151
Syx8	Syntaxin 8	CG4109
CG9705	-	CG9705
CG13024	-	CG13024
CG9951	-	CG9951
CG6664	-	CG6664
CG7728	-	CG7728
CG7707	-	CG7707
CG6479	-	CG6479
CG7580	-	CG7580
Edc3	Enhancer of decapping 3	CG6311
CG5577	-	CG5577
CG16775	-	CG16775
CG14353	-	CG14353
CG13698	-	CG13698
mRpS26	mitochondrial ribosomal protein S26	CG7354
Indy	I'm not dead yet	CG3979
CG11637	-	CG11637
CG18135	-	CG18135
Mkp3	Mitogen-activated protein kinase phosphatase 3	CG14080
CG14100	-	CG14100
CG9368	-	CG9368
CG7757	-	CG7757
CG14185	-	CG14185
CG6981	-	CG6981
Spn77Bc	Serpin 77Bc	CG6289
mRpL15	mitochondrial ribosomal protein L15	CG5219
CG5059	-	CG5059
CG4825	-	CG4825
CG4365	-	CG4365
CG10585	-	CG10585
Neu2	Neu2	CG7204
Cdk12	-	CG7597
CG7370	-	CG7370
CG6838	-	CG6838
CG14451	-	CG14451
CG14450	-	CG14450
CG11367	-	CG11367

slif	slimfast	CG11128
CG12768	-	CG12768
Mes2	Mes2	CG11100
nAcRα-80B	nicotinic Acetylcholine Receptor α 80B	CG12414
CG9795	-	CG9795
CG11739	-	CG11739
Spargel	Spargel	CG9809
CG1074	-	CG1074
CG9804	-	CG9804
CG1129	-	CG1129
CG14657	-	CG14657
CG1115	-	CG1115
Mms19	Mms19	CG12005
CG12173	-	CG12173
elm	ethanol sensitive with low memory	CG2185
CG2023	-	CG2023
CG10286	-	CG10286
Zif	Zinc-finger protein	CG10267
CG1943	-	CG1943
CG2656	-	CG2656
CG1227	-	CG1227
Sp7	Serine protease 7	CG3066
CG2641	-	CG2641
CG10092	-	CG10092
CG10445	-	CG10445
CD98hc	CD98 heavy chain	CG2791
CG3223	-	CG3223
CG2747	-	CG2747
CG7800	-	CG7800
Coq2	Coenzyme Q biosynthesis protein 2	CG9613
CG8116	-	CG8116
Tcp-1η	Tcp-1η	CG8351
CG9837	-	CG9837
CG9821	-	CG9821
CG8379	-	CG8379
CG11966	-	CG11966
Kdm2	Lysine (K)-specific demethylase 2	CG11033
CG9362	-	CG9362
CG8149	-	CG8149

JHDM2	JmjC domain-containing histone demethylase 2	CG8165
mura	murashka	CG9381
SpdS	Spermidine Synthase	CG8327
CG9492	-	CG9492
Pnn	Pinin	CG8383
Teh1	tipE homolog 1	CG12806
CG12814	-	CG12814
CG6293	-	CG6293
CG12818	-	CG12818
CG14691	-	CG14691
Art1	Arginine methyltransferase 1	CG6554
CG4089	-	CG4089
CG17734	-	CG17734
Ranbp9	Ranbp9	CG5252
fabp	fatty acid bindin protein	CG6783
CG14711	-	CG14711
CG10005	-	CG10005
Tk	Tachykinin	CG14734
KLHL18	-	CG3571
MBD-R2	MBD-R2	CG10042
CG5538	-	CG5538
Paip2	polyA-binding protein interacting protein 2	CG12358
CG7966	-	CG7966
CG8449	-	CG8449
CG9813	-	CG9813
CG8870	-	CG8870
CG9796	-	CG9796
omd	oocyte maintenance defects	CG9591
CG9602	-	CG9602
CG14363	-	-
CG9925	-	CG9925
Cyp6d5	Cyp6d5	CG3050
Npc2b	Niemann-Pick type C-2b	CG3153
CG9920	-	CG9920
CG12402	-	CG12402
CG14854	-	CG14854
CG8087	-	CG8087
CG6904	-	CG6904
CG6236	-	CG6236

CG6218	-	CG6218
CG9593	-	CG9593
CG5903	-	CG5903
CG17556	-	CG17556
CG3995	-	CG3995
alt	aluminum tubes	CG18212
CG7379	-	CG7379
CG14315	-	CG14315
CG7714	-	CG7714
CG14291	-	CG14291
CG5555	-	CG5555
CG16718	-	CG16718
CG5466	-	CG5466
CG17838	-	CG17838
CG5630	-	CG5630
Oga	O-GlcNAcase	CG5871
Nelf-A	Negative elongation factor A	CG5874
CG5892	-	CG5892
CG6028	-	CG6028
CG7054	-	CG7054
CG7046	-	CG7046
CG5346	-	CG5346
CG4449	-	CG4449
wda	will decrease acetylation	CG4448
CG10164	-	CG10164
CG10184	-	CG10184
CG10365	-	CG10365
RanBP3	Ran binding protein 3	CG10225
CG10214	-	CG10214
Mta70	Mta70 homologue	CG5933
CG6178	-	CG6178
CG5789	-	CG5789
CG6695	-	CG6695
CG13630	-	CG13630
CG11791	-	CG11791
Mocs2	Molybdopterin synthase 2	CG10238
CG11857	-	CG11857
CHKov2	CHKov2	CG10675
CG5112	-	CG5112

CG6425	-	CG6425
CG5521	-	CG5521
CG14253	-	CG14253
gb	genderblind	CG6070
mrt	martik	CG3361
CG3368	-	CG3368
Moca-cyp	Moca-cyp	CG1866
CG14512	-	CG14512
CG14516	-	CG14516
CG11897	-	CG11897
CG2310	-	CG2310
CG7582	-	CG7582
eIF2B-α	eIF2B-α	CG7883
CG15523	-	CG15523
CG9737	-	CG9737
CG18404	-	CG18404
mRpS18C	mitochondrial ribosomal protein S18C	CG9688
CG1746	-	CG1746
CG11334	-	CG11334
CG2118	-	CG2118
CG2219	-	CG2219
yellow-h	yellow-h	CG1629
CG1674	-	CG1674
Asator	Asator	CG11533
CG1970	-	CG1970
fd102C	forkhead domain 102C	CG11152
Sox102F	Sox102F	CG11153
CG17163	-	CG17163
CG17162	-	CG17162
CG17159	-	CG17159
CR40282	-	CR40282
JYalpha	JYalpha	CG17923
vav	vav	CG7893
Trx-2	thioredoxin-2	CG31884
tara	taranis	CG6889
pont	pontin	CG4003
raps	rapsynoid	CG5692
lectin-28C	lectin-28C	CG7106

lectin-24Db	lectin-24Db	CG2958
kat80	katanin 80	CG13956
Ugt86Da	Ugt86Da	CG18578
Spt5	Spt5	CG7626
SF2	SF2	CG6987
SC35	SC35	CG5442
Nhe2	Na+/H+ hydrogen exchanger 2	CG9256
Jafrac1	thioredoxin peroxidase 1	CG1633
Gclc	Glutamate-cysteine ligase catalytic subunit	CG2259
Ephrin	Ephrin	CG1862
TRAM	TRAM	CG11642
CG3711	-	CG3711
CG11638	-	CG11638
G9a	G9a	CG2995
CG5254	-	CG5254
CG32795	-	CG32795
boi	brother of ihog	CG32796
CG14050	-	CG14050
Dip3	Dorsal interacting protein 3	CG12767
CG15864	-	CG15864
CG15496	-	-
CG12848	-	CG12848
CG15234	-	-
CG11373	-	CG11373
CG14663	-	-
CG12483	-	CG12483
CG11930	-	-
Nplp4	Neuropeptide-like precursor 4	CG15361
CG14350	-	-
CG16906	-	-
Prosap	Prosap	CG30483
CoVIIc	Cytochrome c oxidase subunit VIIc	CG2249
CG17977	-	CG17977
dpr6	dpr6	CG14162
CG8620	-	CG8620
CR32658	-	CR32658
CG14809	-	-
CG13757	-	-
CG12643	-	CG12643

CG13130	-	CG13130
CG4440	-	CG4440
CG6115	-	CG6115
CG13302	-	-
wun2	wunen-2	CG8805
tai	taiman	CG13109
rols	rolling pebbles	CG32096
comm2	comm2	CG7554
armi	armitage	CG11513
TepII	Thiolester containing protein II	CG7052
Rheb	Rheb	CG1081
HDAC4	HDAC4	CG1770
Gr93a	Gustatory receptor 93a	CG13417
Cct1	CTP:phosphocholine cytidylyltransferase 1	CG1049
olf186-F	olf186-F	CG11430
cpx	complexin	CG32490
TAS	Telomeric Associated Sequences	-
snRNA:U2:34ABc	small nuclear RNA U2 at 34ABc	CR33788
tral	trailer hitch	CG10686
CG18819	-	-
CG18823	-	CG18823
CR18854	-	CR18854
CG18858	-	CG18858
CG18769	-	CG18769
CG17376	-	CG17376
CG13713	-	CG13713
PP2A-B'	PP2A-B'	CG7913
Chrac-14	Chrac-14	CG13399
CG10251	-	CG10251
Fsn	-	CG4643
AP-2σ	AP-2σ	CG6056
snRNA:U5:38ABa	snRNA:U5:38ABa	CR32881
MESK2	Misexpression suppressor of KSR 2	CG15669
bchs	blue cheese	CG14001
CG12084	-	CG12084
vir-1	virus-induced RNA 1	CG31764
pygo	pygopus	CG11518
dome	domeless	CG14226
Roc2	Roc2	CG8998

Ilp6	Insulin-like peptide 6	CG14049
Chro	Chromator	CG10712
Scim9	Sensitized chromosome inheritance modifier 9	-
Scim8	Sensitized chromosome inheritance modifier 8	-
Scim7	Sensitized chromosome inheritance modifier 7	-
Scim6	Sensitized chromosome inheritance modifier 6	-
Scim5	Sensitized chromosome inheritance modifier 5	-
Scim4	Sensitized chromosome inheritance modifier 4	-
Scim37	Sensitized chromosome inheritance modifier 37	-
Scim36	Sensitized chromosome inheritance modifier 36	-
Scim35	Sensitized chromosome inheritance modifier 35	-
Scim34	Sensitized chromosome inheritance modifier 34	-
Scim33	Sensitized chromosome inheritance modifier 33	-
Scim32	Sensitized chromosome inheritance modifier 32	-
Scim30	Sensitized chromosome inheritance modifier 30	-
Scim29	Sensitized chromosome inheritance modifier 29	-
Scim28	Sensitized chromosome inheritance modifier 28	-
Scim27	Sensitized chromosome inheritance modifier 27	-
Scim26	Sensitized chromosome inheritance modifier 26	-
Scim25	Sensitized chromosome inheritance modifier 25	-
Scim24	Sensitized chromosome inheritance modifier 24	-
Scim23	Sensitized chromosome inheritance modifier 23	-
Scim22	Sensitized chromosome inheritance modifier 22	-
Scim21	Sensitized chromosome inheritance modifier 21	-
Scim20	Sensitized chromosome inheritance modifier 20	-
Scim18	Sensitized chromosome inheritance modifier 18	-
Scim17	Sensitized chromosome inheritance modifier 17	-
Scim16	Sensitized chromosome inheritance modifier 16	-
Scim15	Sensitized chromosome inheritance modifier 15	-
Scim14	Sensitized chromosome inheritance modifier 14	-
Scim13	Sensitized chromosome inheritance modifier 13	-
Scim12	Sensitized chromosome inheritance modifier 12	-
Scim11	Sensitized chromosome inheritance modifier 11	-
Scim10	Sensitized chromosome inheritance modifier 10	-
Pak3	Pak3	CG14895
bwa	brain washing	CG13969
CHKov1	CHKov1	CG10618
yuri	yuri gagarin	CG31732
CG12699	-	CG12699

p38c	p38c	CG33338
CR14033	-	CR14033
gem	gemini	CG30011
CG30089	-	CG30089
CCHa1r	CCHamide-1 receptor	CG30106
CG30118	-	CG30118
CG30122	-	CG30122
Hil	Hillarin	CG30147
Brca2	Breast cancer 2, early onset homolog	CG30169
CG30183	-	CG30183
CG30286	-	CG30286
CG30372	-	CG30372
CG30379	-	CG30379
CG30389	-	CG30389
CG30401	-	CG30401
Atf-2	Activating transcription factor-2	CG30420
CG30421	-	CG30421
CG30438	-	CG30438
CG30460	-	CG30460
CG30463	-	CG30463
CG30497	-	CG30497
CG31064	-	CG31064
CG31108	-	CG31108
CG31140	-	CG31140
Rpb7	Rpb7	CG31155
CG31176	-	CG31176
CG31191	-	CG31191
CG31211	-	CG31211
Naam	Nicotinamide amidase	CG31216
CG31220	-	CG31220
koko	kokopelli	CG31232
CG31235	-	CG31235
CG31324	-	CG31324
CG31344	-	CG31344
Unc-115a	-	CG31352
Jupiter	Jupiter	CG31363
CG31445	-	CG31445
CG31510	-	CG31510
CG31522	-	CG31522

CG31523	-	CG31523
CG31547	-	CG31547
CG31619	-	CG31619
CG31626	-	CG31626
CG31635	-	CG31635
stai	stathmin	CG31641
CG31673	-	CG31673
CG31678	-	CG31678
CG31690	-	CG31690
CG31694	-	CG31694
CG31715	-	CG31715
RluA-1	RluA-1	CG31719
Trim9	Trim9	CG31721
CG31729	-	CG31729
CG31793	-	CG31793
CG31814	-	CG31814
CG31974	-	CG31974
CG32043	-	CG32043
A2bp1	Ataxin-2 binding protein 1	CG32062
S-Lap4	Sperm-Leucylaminopeptidase 4	CG32064
CG32109	-	CG32109
tRNA:CR32123:Ψ	Transfer RNA:CR32123:pseudogene	CR32123
CG32137	-	CG32137
Krn	Keren	CG32179
CG32262	-	CG32262
CG32365	-	CG32365
shep	alan shepard	CG32423
CG32425	-	CG32425
CG32447	-	CG32447
CG32452	-	CG32452
CG32473	-	CG32473
CG32479	-	CG32479
Sk2	Sphingosine kinase 2	CG32484
CG32529	-	CG32529
CG32541	-	CG32541
CG32548	-	CG32548
CG32590	-	CG32590
dpr8	dpr8	CG32600
βNACtes3	-	CG32601

CG32635	-	CG32635
CG32638	-	CG32638
CG32640	-	CG32640
Drak	Death-associated protein kinase related	CG32666
Atg8a	Autophagy-specific gene 8a	CG32672
CG32676	-	CG32676
CG32699	-	CG32699
CG32700	-	CG32700
CG32772	-	CG32772
CG32792	-	CG32792
mRpL27	mitochondrial ribosomal protein L27	CG33002
CG33096	-	CG33096
Rtnl1	Rtnl1	CG33113
CG33129	-	CG33129
CG33144	-	CG33144
CG33156	-	CG33156
CG33169	-	CG33169
CG33181	-	CG33181
Kdm4B	Histone demethylase 4B	CG33182
CG33203	-	CG33203
Mical	Molecule interacting with CasL	CG33208
CG33276	-	CG33276
CG33293	-	CG33293
CG33309	-	CG33309
CheB42a	Chemosensory protein B 42a	CG33348
PNUTS	PNUTS	CG33526
Ddr	Discoidin domain receptor	CG33531
Nipped-A	Nipped-A	CG33554
form3	formin 3	CG33556
mim	missing-in-metastasis	CG33558
CG33988	-	CG33988
fd3F	forkhead domain 3F	CG12632
His2B:CG17949	His2B:CG17949	CG17949
CG33671	-	CG33671
CG33672	-	CG33672
Ube3a	Ubiquitin protein ligase E3A	CG6190
Lasp	Lasp	CG3849
GstE6	Glutathione S transferase E6	CG17530
GstE3	Glutathione S transferase E3	CG17524

CG33713	-	CG33713
CG33714	-	CG33714
CG33691	-	CG33691
stg1	stargazin-like protein	CG33670
snoRNA:185	snoRNA:185	CR33930
l(2)309	lethal (2) 309	-
dbr	debra	CG11371
akirin	akirin	CG8580
pncr011:3L	putative noncoding RNA 011:3L	CR33947
lobo	lost boys	CG34110
CG34164	-	CG34164
inaF-C	inaF-C	CG34321
inaF-A	inaF-A	CG34322
CG34357	-	CG34357
Shroom	Shroom	CG34379
CG34383	-	CG34383
CG34384	-	CG34384
Epac	Epac	CG34392
nub	nubbin	CG34395
CG34396	-	CG34396
pan	pangolin	CG34403
spri	sprint	CG34414
CG34417	-	CG34417
sif	still life	CG34418
Hmx	H6-like-homeobox	CG34419
Snoo	Sno oncogene	CG34421
KH1	KH1	CG3561
del	deadlock	CG9252
Myo31DF	Myosin 31DF	CG7438
Tpi	Triose phosphate isomerase	CG2171
Sec61α	Sec61α	CG9539
Orct2	Organic cation transporter 2	CG13610
sra	sarah	CG6072
lap	like-AP180	CG2520
Mer	Merlin	CG14228
mib2	mind bomb 2	CG17492
l(2)37Cd	lethal (2) 37Cd	CG10563
l(2)37Cg	lethal (2) 37Cg	CG10685
Spt-I	Serine palmitoyltransferase subunit I	CG4016

jing	jing	CG9397
shrb	shrub	CG8055
Spf45	Spf45	CG17540
frtz	fritz	CG17657
stops	slow termination of phototransduction	CG31006
Egm	Enigma	CG9006
chinmo	Chronologically inappropriate morphogenesis	CG31666
step	steppke	CG11628
amn	amnesiac	CG11937
stmA	stambha A	CG8739
CG17078	-	CG17078
tlk	Tousled-like kinase	CG34412
kis	kismet	CG3696
sls	sallimus	CG1915
egg	eggless	CG12196
l(3)neo38	lethal (3) neo38	CG6930
rg	rugose	CG6775
tal	tarsal-less	-
bbg	big bang	CG42230
e(y)3	enhancer of yellow 3	CG12238
rdo	reduced ocelli	CG15151
puc	puckered	CG7850
CG42232	-	CG42232
β-Spec	β Spectrin	CG5870
CG5802	-	CG5802
gish	gilgamesh	CG6963
Pros35	Proteasome 35kD subunit	CG4904
26-29-p	26-29kD-proteinase	CG8947
CG42238	-	CG42238
CG42239	-	CG42239
Sema-5c	Semaphorin-5c	CG5661
Pgk	Phosphoglycerate kinase	CG3127
CG42248	-	CG42248
futsch	futsch	CG34387
Ndae1	Na+-driven anion exchanger 1	CG42253
CG42258	-	CG42258
mnb	minibrain	CG42273
α-Man-I	α Mannosidase I	CG42275
ome	omega	CG42280

bun	bunched	CG42281
prom	prominin	CG42310
Doa	Darkener of apricot	CG42320
CG42321	-	CG42321
Pde1c	Phosphodiesterase 1c	CG42325
CG42327	-	CG42327
comm3	comm3	CG42334
brp	bruchpilot	CG42344
Mob2	Mob2	CG11711
Mob4	-	CG3403
CG42351	-	CG42351
crb	crumbs	CG6383
CG42353	-	CG42353
CG42354	-	CG42354
CG42388	-	CG42388
CG42389	-	CG42389
RhoGEF3	-	CG42378
wech	wech	CG42396
mmy	mummy	CG9535
ab	abrupt	CG4807
pzg	putzig	CG7752
bora	aurora borealis	CG6897
CG42399	-	CG42399
out	outsiders	CG8062
inaF-B	inaF-B	CG42447
l(2)35Cc	lethal (2) 35Cc	CG15266
Mppe	Metallophosphoesterase	CG8889
nab	nab	CG33545
rump	rumpelstiltskin	CG9373
KG01932	-	-
CG42514	-	CG42514
CG42518	-	CG42518
Su(var)3-3	Suppressor of variegation 3-3	CG17149
Pbp49	PSEA-binding protein 49kD	CG42515
MED9	Mediator complex subunit 9	CG42517
GABA-B-R1	metabotropic GABA-B receptor subtype 1	CG15274
CG12163	-	CG12163
CG32714	-	-
dl	dorsal	CG6667

CR42549	-	CR42549
wisp	wispy	CG15737
p120ctn	Adherens junction protein p120	CG17484
inaF-D	inaF-D	CG42563
dnr1	defense repressor 1	CG12489
Ir76a	Ionotropic receptor 76a	CG42584
cuff	cutoff	CG13190
tay	tay bridge	CG9056
Rbp6	RNA-binding protein 6	CG32169
Atg1	Autophagy-specific gene 1	CG10967
Baldspot	Baldspot	CG3971
pic	piccolo	CG7769
CG42594	-	CG42594
RfC4	Replication factor C subunit 4	CG14999
para	paralytic	CG9907
stj	straightjacket	CG12295
Sytα	Synaptotagmin α	CG5559
Xrp1	-	CG17836
Alh	Alhambra	CG1070
Hr39	Hormone receptor-like in 39	CG8676
inaE	inactivation no afterpotential E	CG33174
rgn	regeneration	CG6014
Fhos	-	CG42610
SelD	Selenide,water dikinase	CG8553
grp	grapes	CG17161
verm	vermiform	CG8756
CG42630	-	CG42630
mtTFB1	Mitochondrial Transcription Factor B1	CG42631
IntS6	Integrator 6	CG3125
α-Est5	α-Esterase-5	CG1089
didum	dilute class unconventional myosin	CG2146
DhpD	Dihydropterin deaminase	CG18143
CG3638	-	CG3638
trol	terribly reduced optic lobes	CG33950
RhoGAP18B	RhoGAP18B	CG42274
CG42666	-	CG42666
rdgA	retinal degeneration A	CG42667
CG42674	-	CG42674
Thor	Thor	CG8846

CG42678	-	CG42678
Lmpt	Limpet	CG42679
CG42684	-	CG42684
CG42686	-	CG42686
RpS26	Ribosomal protein S26	CG10305
nej	nejire	CG15319
mbl	muscleblind	CG33197
Axud1	-	CG4272
nemy	no extended memory	CG8776
Gyc76C	Guanylyl cyclase at 76C	CG42636
gce	germ cell-expressed bHLH- PAS	CG42739
Dbx	Dbx	CG42234
bru	brunelleschi	CG2478
SmE	Small ribonucleoprotein particle protein SmE	CG18591
Trf2	TATA box binding protein- related factor 2	CG18009
kcc	kazachoc	CG5594
Dhc64C	Dynein heavy chain 64C	CG7507
LanB1	LanB1	CG7123
CG42748	-	CG42748
pico	pico	CG11940
Msp-300	Muscle-specific protein 300	CG42768
aPKC	atypical protein kinase C	CG42783
whd	withered	CG12891
sdt	stardust	CG32717
l(2)35Bc	lethal (2) 35Bc	CG4103
osa	osa	CG7467
dikar	dikar	CG42799
kdn	knockdown	CG3861
CG42817	-	CG42817
f	forked	CG42864
gho	ghost	CG10882
magu	magu	CG2264
Tl	Toll	CG5490
CG43066	-	CG43066
nrm	neuromusculin	CG43079
Vha44	+ Vacuolar H ATPase 44kD C subunit	CG8048
VhaAC45	+ Vacuolar H ATPase accessory protein AC45	CG8029
Mi-2	-	CG8103
vas	vasa	CG43081

cic	capicua	CG43122
sec8	sec8	CG2095
pyd	polychaetoid	CG43140
CG43143	-	CG43143
Lin29	-	CG2052
CG43154	-	CG43154
Src64B	Src oncogene at 64B	CG7524
Imp	IGF-II mRNA-binding protein	CG1691
CG11727	-	CG11727
Fas1	Fasciclin 1	CG6588
lute	lute	CG43226
rdx	roadkill	CG12537
cnc	cap-n-collar	CG43286
lawc	leg arista wing complex	CG32711
Ca-P60A	Calcium ATPase at 60A	CG3725
CG43345	-	CG43345
CG43346	-	CG43346
psq	pipsqueak	CG2368
bft	bereft	CR42938
bin3	bicoid-interacting protein 3	CG8276
Galt	Galactose-1-phosphate uridylyltransferase	CG9232
Hk	Hyperkinetic	CG43388
Coop	Corepressor of Pangolin	CG1621
Mocs1	Molybdenum cofactor synthesis 1 ortholog	CG33048
vnc	variable nurse cells	CG11989
chas	chascon	CG32556
scrib	scribbled	CG43398
CG31688	-	CG31688
hts	hu li tai shao	CG43443
CG43444	-	CG43444
hppy	happyhour	CG7097
CG43462	-	CG43462
nclb	no child left behind	CG6751
Lpin	Lipin	CG8709
CG43674	-	CG43674
fok	fledgling of Klp38B	CG43690
Hr4	Hr4	CG43692
CG34334	-	CG34334
Smr	Smrter	CG4013

sick	sickie	CG43720
