# Supplementary material for: Identification of Genes Underlying Hypoxia Tolerance in Drosophila by a P-element Screen
Source: G3 (Bethesda). 2012 Oct 1;2(10):1169–78. doi: 10.1534/g3.112.003681 (PMC3464109; doi:10.1534/g3.112.003681)
Supplement: Supporting Information [file supp_2.10.1169_TableS1.pdf]

Table S1 Primer sequences of genes tested by real time PCR

GENE NAME	PRIMER SEQUENCE
Sec8-L	TTGCCATCATAGTGGAGTCG
Sec8-R	CGTTGAGCAACTCTGTTTGG
CG10700-L	ACATTGGGCGAGTTGATAGG
CG10700-R	CCCAAAATCCTGATGATTCC
Osa-L	CATCTCAAAACAGCCACTCG
Osa-R	CTTCCAGGTCTTGCTCTTGG
mRpS18B-L	CAACATTACCCGGAATAGCC
mRpS18B-R	CCGCATTATTTTCTTCAGTCG
l(3)mbn-L	TGTGACAACGACTCCAAAGG
l(3)mbn-R	TAGGAAACAACCGGAACAGG
Atg1-L	CTGAAAATTGCGGATTTTGG
Atg1-R	CGCCATATACATGGGAGAGC
CG5235-L	CGCTATCCCGAGTTTACAGC
CG5235-R	AATTGTGAACTTCCCGAACG
Cpa-L	CGAAGTGTTCAACGATGTGC
Cpa-R	CTTGTTGTATTGGGCGAAGG
Mys45A-L	CACCTCGCTCAAGTTCTTCC
Mys45A-R	TAAGGTCCACCTCGTTCTCG
Iqf-L	GGTTGGCTGCAAAGTAATGG
lqf-R	TTTATCAGCCAGCCTTCAGC
tna-L	AGTTCTATCCGGGCAGTGG
tna-R	GCTGGAAACCTTGATTCTGG
Scrib-L	AATAAGCCAGCTGACGAAGC
Scrib-R	CGGCAGATAACCCAAATAGG
Drp1-L	AGGATGTCTCCGACAAGAGC
Drp1-R	TTCCGTGGTCTCGATATTCC
CG6230-L	GTACCCACTCCCAACAATGG
CG6230-R	TGTCTTCCGATCCTCATTCC
CG6860-L	AATACGATTCGCAGCATTCC
CG6860-R	GAGGCAGGAAACAAATCTCG
Rep2-L	GGGTTAGGCTTTCGACTTCC
Rep2-R	AACGTACGTGCTTCCTTTCG
Bgb-1	TGGCTGTCTGGAGTATGACG
Bgb-2	AACTAACGACGCCACATGC
CG17273-L	ACACGTGCATCTGTCTGACC
CG17273-R	GGAAGTGGTCAAGCTTCTCG

CG8116-L	CACTGTGCATCATCGAGACC
CG8116-R	AATATTACGGATGCGGTTGC
CG8180-L	GCAAAGCGAGACAAAAGAGC
CG8180-R	CAAGGAGCCAAGTTTTCTGC
Manf-L	CGACTTCATCAAGCGTATCG
Manf-R	TAGCTAAGTGGGCGTGTTCC
Ci-L	GCAGCGGCTACTGTTTTACC
Ci-R	CCCGTTGGAATAATTGATGG
